# CD4 occupancy triggers sequential pre-fusion conformational states of the HIV-1 envelope trimer with relevance for broadly neutralizing antibody activity

**DOI:** 10.1371/journal.pbio.3000114

**Published:** 2019-01-16

**Authors:** Branislav Ivan, Zhaozhi Sun, Harini Subbaraman, Nikolas Friedrich, Alexandra Trkola

**Affiliations:** Institute of Medical Virology, University of Zurich, Zurich, Switzerland; Centre International de Recherche en Infectiologie (CIRI), FRANCE

## Abstract

During the entry process, the human immunodeficiency virus type 1 (HIV-1) envelope glycoprotein (Env) trimer undergoes a sequence of conformational changes triggered by both CD4 and coreceptor engagement. Resolving the conformation of these transient entry intermediates has proven challenging. Here, we fine-mapped the antigenicity of entry intermediates induced by increasing CD4 engagement of cell surface–expressed Env. Escalating CD4 triggering led to the sequential adoption of different pre-fusion conformational states of the Env trimer, up to the pre-hairpin conformation, that we assessed for antibody epitope presentation. Maximal accessibility of the coreceptor binding site was detected below Env saturation by CD4. Exposure of the fusion peptide and heptad repeat 1 (HR1) required higher CD4 occupancy. Analyzing the diverse antigenic states of the Env trimer, we obtained key insights into the transitions in epitope accessibility of broadly neutralizing antibodies (bnAbs). Several bnAbs preferentially bound CD4-triggered Env, indicating a potential capacity to neutralize both pre- and post-CD4 engagement, which needs to be explored. Assessing binding and neutralization activity of bnAbs, we confirm antibody dissociation rates as a driver of incomplete neutralization. Collectively, our findings highlight a need to resolve Env conformations that are neutralization-relevant to provide guidance for immunogen development.

## Abstract 2

Comprehensive mapping of conformational stages adopted by the HIV‐1 envelope glycoprotein trimer during entry into the cell reveals the preference of broadly neutralizing antibodies for distinct pre-fusion states of the trimer.

## Author summary

The trimeric human immunodeficiency virus type 1 (HIV-1) envelope glycoprotein (Env) mediates HIV-1 entry into its target cells. Entry is initiated by sequential triggering of Env upon interaction with its primary receptor CD4 and a coreceptor on target cells. The ensuing structural rearrangements of the Env trimer bring the viral membrane in close vicinity of the cellular membrane, enabling fusion. Resolving the structural differences between the consecutive conformations Env adopts during the entry process is of high interest, as different antigenic domains are exposed, which may affect the capacity of neutralizing antibodies to bind to Env and inhibit entry. Here, we compared the conformation of unliganded closed Env with the transitional CD4-bound Env forms by studying the antigenicity of cell surface–expressed Env with and without CD4 triggering. We show that incremental triggering by soluble CD4 allows the capture of the full continuum of conformational changes, including events that follow coreceptor interaction. Thus, the setup we introduce here turns a simple binding assay into a powerful tool to study transitional conformation changes in HIV-1 Env. Analyzing the capacity of Env-reactive antibodies to recognize the diverse Env stages, our study reveals novel aspects of the binding preferences of neutralizing antibodies that affect their inhibitory activity.

## Introduction

Human immunodeficiency virus type 1 (HIV-1) envelope glycoprotein (Env) trimers, composed of three gp41-gp120 heterodimers, initiate the virus entry process by binding to the primary receptor CD4 via a high-affinity binding site (referred to as CD4bs) on gp120. CD4 engagement triggers conformational changes that facilitate the binding of a coreceptor [1]. Subsequently, a second wave of conformational changes occurs within the gp41 subunit that leads to the release of the hydrophobic fusion peptide (FP) and finally the fusion of the viral and target cell membranes [2,3]. Important discoveries in recent years have highlighted that native Env is conformationally flexible even in the absence of receptor triggers, shifting dynamically between a closed ground state and activated open states resembling those triggered by CD4 engagement [4,5].

CD4 triggering exposes neutralization-vulnerable epitopes shielded on the native trimer, including the third hypervariable (V3) loop crown and the CD4-induced site (CD4i) [6–8]. Paradoxically, V3 crown and CD4i antibodies are abundant in HIV-1 infection but have only a weak or no detectable neutralization activity at all because of their limited capacity to access their epitopes on the closed Env trimer [9–12]. In contrast, the rare broadly neutralizing antibodies (bnAbs) that are elicited [13] overcome the shielding restriction and neutralize a wide spectrum of global HIV-1 strains [14]. Although the capacity to bind to the closed (pre-CD4-bound) Env is generally thought to be critical for HIV-1 neutralizing antibody (nAb) activity [4,15–17], the precise modes of action differ between individual nAbs and include direct interference with CD4 or coreceptor engagement [18,19], arrest of Env in the ground state [4,5,20], or acceleration of trimer decay by capturing Env in an activated state [4,21–23].

Despite detailed structural information on multiple Env conformations [2,24–26], certain aspects of the entry process have not yet been fully unraveled. Soluble Env trimers, the basis of much of our current knowledge of Env structure, do not fully represent the wild-type membrane-embedded Env trimer [27–32]. Structural information on the native CD4-bound Env is comparatively limited and stems from a small number of lower-resolution cryo–electron tomography (cryo-ET) reconstructions [33,34]. Theoretically, each of the three gp120 protomers of the Env trimer has the capacity to bind CD4 and the coreceptor. However, how many CD4 and coreceptor molecules need to interact with an Env trimer in order for gp41 to undergo the necessary conformational rearrangements remains unclear [35]. A requirement of fewer than three CD4 proteins per Env has been proposed based on functional assays with mixed Env trimers that contained one or two CD4 binding–deficient gp120 subunits [35–41]. Yet, only one structural study so far has provided information on an Env conformation with partial CD4 occupancy using highly stabilized soluble Env trimers [42].

Here, we developed a strategy to investigate the antigenicity of native, membrane-embedded HIV-1 Env upon triggering with soluble CD4 (sCD4). The “on-cell sCD4-triggering assay” we introduce allows fine antigenic mapping of the conformational dynamics of the full-length Env trimer and resolution of dynamic changes in the antigenic landscape upon receptor triggering. Assessing the capacity of bnAbs in recognizing differentially CD4-triggered Env intermediates, we obtained novel insights into the interdependencies of bnAb binding and neutralization.

## Results

### Induction of sequential, antigenically distinct HIV-1 Env states by sCD4 triggering

**Fig 1:**
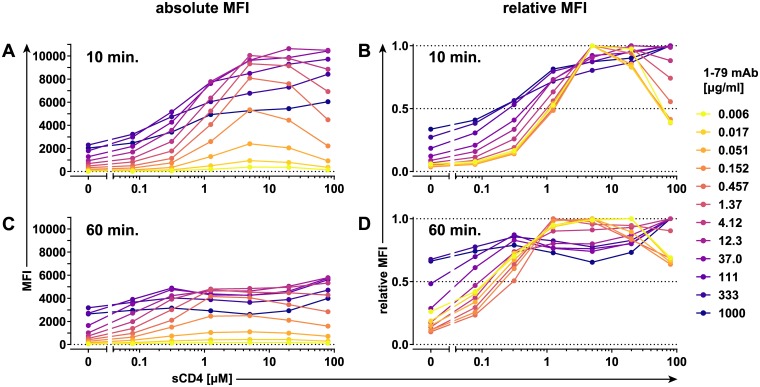
Triggering conformational changes in cell surface–expressed BaL.01 Env by sCD4. HEK 293T cells expressing BaL.01 Env were stained with indicated concentrations of V3 crown-directed mAb 1–79 in the presence of increasing concentrations of two-domain sCD4 for 10 minutes (A, B) or 60 minutes (C, D). The amount of antibody bound to the cell surface was quantified by flow cytometry. Dead cells were excluded from analysis, and background signal from MuLV Env–expressing control cells was subtracted. MFI staining curves (A, C) or MFI staining curves normalized to their maxima (relative MFI staining curves) (B, D) are plotted for every antibody concentration. Histogram plots of the event distributions underlying the above MFI values are depicted in S1 Fig. Data represent a single experiment. Env, envelope glycoprotein; HEK, human embryonic kidney; mAb, monoclonal antibody; MFI, mean of fluorescence intensity; MuLV, murine leukemia virus; sCD4, soluble CD4; V3, third hypervariable.

Recent discoveries have highlighted the intrinsic flexibility of the unliganded HIV-1 Env trimer complex [4,5]. Here, we sought to derive detailed information on the shifts in epitope exposure in CD4-unbound and CD4-bound forms of native, membrane-embedded HIV-1 Env by employing an experimental procedure we refer to as on-cell sCD4 triggering assay. The assay assesses the capacity of Env-directed monoclonal antibodies (mAbs) to bind to Env transiently expressed on cells in the presence of increasing concentrations of sCD4. Binding of mAbs (S1 Table) to the differentially CD4-triggered Env was assessed by flow cytometry. We used mAbs as indicators of specific conformational states and therefore sought to avoid conditions in which the mAbs themselves would influence Env conformation. To define the appropriate conditions, we titrated each individual mAb over a wide concentration range in the absence and presence of varying concentrations of sCD4 using cells expressing the Env of the Tier 1B subtype B strain BaL.01 (Fig 1, S1, S3, S4 and S5 Figs). As exemplified by the V3 crown-specific mAb 1-79 (Fig 1 and S1 Fig), the sCD4-enhanced exposure of gp120 V3 crown on BaL.01 Env is readily detectable after 10 minutes and increases up to a mAb dose of approximately 10 μg/ml for all probed sCD4 concentrations (Fig 1A). Whereas 1-79 concentrations higher than about 10 μg/ml resulted in increased staining of CD4-unbound and low-level sCD4-triggered Env, the signal in presence of medium and high sCD4 concentrations started to decline (Fig 1A), likely reflecting the onset of gp120 shedding, the release of gp120 from Env trimers [21]. We next normalized all staining curves of 1-79 to their individual maxima (1.0 = highest value measured at any particular sCD4 concentration) (Fig 1B). At lower 1-79 mAb concentrations (0.006–0.152 μg/ml), the normalized curves converge and tightly overlap (Fig 1B). We refer to this as the basal epitope exposure curve of a mAb. The 1-79 basal epitope exposure curve rises along with increasing sCD4 concentration, peaks at approximately 5 μM sCD4, and decreases thereafter (Fig 1B). The native Env is known to continuously sample different conformations [5]. Antibodies specific for a conformation can bind and stabilize it, which is referred to as “conformation capture.” As the conformational states are often short-lived [5], the antibody concentration is expected to be a limiting factor in recognition. Employing the basal epitope exposure curves rather than staining curves obtained at higher antibody concentrations is thus crucial if native and not antibody-enforced conformational states are to be investigated. Extending the triggering/staining step to 60 minutes (Fig 1C and 1D) revealed an increase in gp120 shedding, with maximum 1–79 staining of sCD4-triggered Envs decreasing by up to 50% (Fig 1C). The increased staining of CD4-unbound and low-level sCD4-triggered Envs highlighted that elongated reaction times provide more opportunities for conformation capture (Fig 1C). These time- and antibody concentration–dependent effects eventually amount to a flattening of the basal epitope exposure curve, obscuring its otherwise distinct peak (Fig 1D).

**Fig 2:**
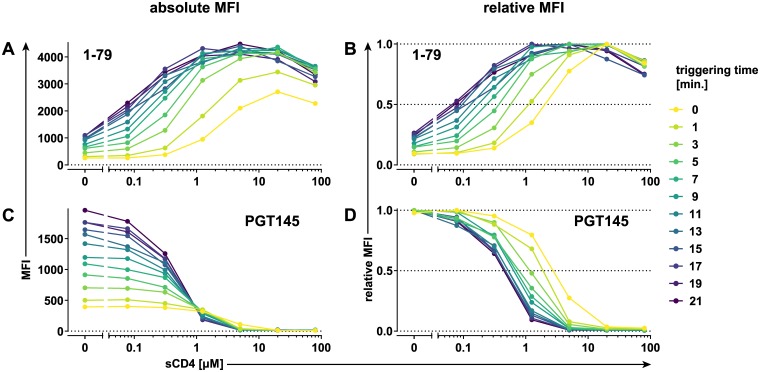
Time resolution of on-cell sCD4 triggering to define basal epitope exposure curves. Time resolution of on-cell sCD4 triggering of BaL.01 Env. Binding efficacy of V3 crown mAb 1–79 (A, B) and V2 apex bnAb PGT145 recognizing a quaternary epitope (C, D) (both at 0.1 μg/ml) in response to increasing sCD4 at different time intervals. MFI staining curves (A, C) or MFI staining curves normalized to their maxima (relative MFI staining curves) (B, D) derived as described in Fig 1 are plotted for all individual time points. Histogram plots of the event distributions underlying the above MFI values are depicted in S2 Fig. Data represent a single experiment. bnAb, broadly neutralizing antibody; Env, envelope glycoprotein; mAb, monoclonal antibody; MFI, mean of fluorescence intensity; sCD4, soluble CD4; V2, second hypervariable; V3, third hypervariable.

To gain a better insight into the time development of the basal epitope exposure curve, we performed the on-cell sCD4 triggering assay with 1–79 mAb and second hypervariable (V2) loop apex mAb PGT145 at a low concentration (0.1 μg/ml) and stopped the reaction at frequent intervals (Fig 2 and S2 Fig). Although the staining intensity by both mAbs increased during the 20-minute observation time, they were influenced differently by the sCD4 concentration (Fig 2A and 2C). The PGT145 staining curves converged to a singular shape after approximately 10 minutes (Fig 2D). In contrast, the 1–79 staining curves quickly reached maximum staining around the 5 μM sCD4 peak while continuing to increase at low or no sCD4 throughout the experiment (Fig 2B). Therefore, although one can limit the influence of conformation capture by minimizing the detection mAb concentration, the on-cell triggering assay is inherently dynamic, and the conformation capture will inevitably manifest itself in the varying mAb binding on-rates depending on the sCD4 concentration. Nevertheless, after a minimum incubation time of approximately 5 minutes, which is necessary to establish a clear basal epitope exposure curve maximum, the sCD4 concentration corresponding to the curve maximum remains stable even as the signal continues to increase (Fig 2B and 2D). It is this property of the basal epitope exposure curve development that allows for the retrieval of information on the conformational preference of antibodies by comparing the positions of their respective basal epitope exposure curve maxima. Based on these analyses, we chose an optimal triggering/staining step of 20 minutes because we found no evidence for increased signal loss, and thus gp120 shedding, versus shorter incubation times. In addition, the higher signal provided by the longer, 20-minute incubation step allowed us to derive basal epitope exposure curves even for weakly staining mAbs and inhibitors (S3 and S4 Figs). The basal epitope exposure curves retrieved in independent experiments showed an identical pattern (S5 Fig), allowing a robust readout also from single experiments.

**Fig 3:**
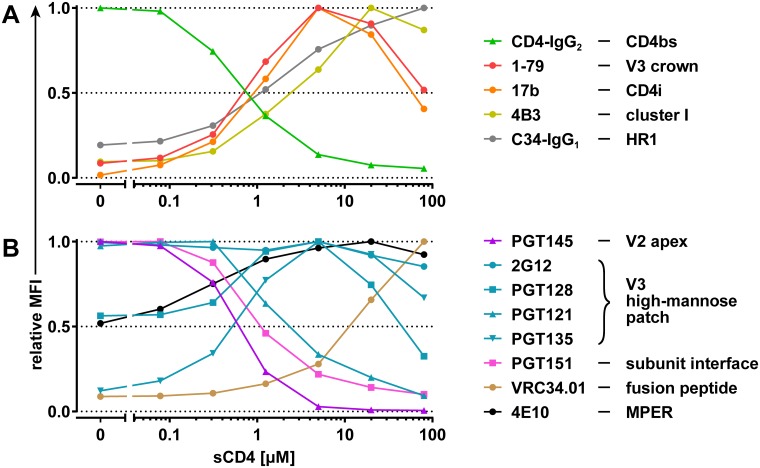
BaL.01 Env basal epitope exposure curves of mAbs and inhibitors. On-cell sCD4 triggering of BaL.01 Env with a 20-minute triggering step. Basal epitope exposure curves for wnAbs/nnAbs, CD4-IgG_2_, and C34-IgG_1_ depicted in (A) and bnAbs depicted in (B) were selected from the relative MFI staining curves of each individual antibody/inhibitor (S3 Fig) based on the following criteria: lowest concentration of the tested antibody/inhibitor that yields at least a 10-fold-higher MFI signal over MuLV background at the peak. Histogram plots of the event distributions underlying the basal epitope exposure curves are depicted in S4 Fig. bnAb, broadly neutralizing antibody; CD4bs, CD4 binding site; CD4i, CD4-induced site; Env, envelope glycoprotein; HR1, heptad repeat 1; mAb, monoclonal antibody; MFI, mean of fluorescence intensity; MPER, membrane-proximal external region; MuLV, murine leukemia virus; nnAb, nonneutralizing antibody; sCD4, soluble CD4; V2, second hypervariable; V3, third hypervariable; wnAb, weakly neutralizing antibody.

We acquired basal epitope exposure curves of BaL.01 Env for a panel of anti-Env mAbs directed against antigenic sites on Env that are exposed upon CD4 engagement (Fig 3A, S3 and S4 Figs). This included mAbs directed to the V3 crown, the CD4i domain on gp120, and the immunodominant cluster I of gp41 [7,8]. To probe the accessibility of the heptad repeat 1 (HR1) binding groove, which depends on CD4 engagement and is linked with a fusion-competent activated Env state that allows HR1–heptad repeat 2 (HR2) interaction [43,44], we used the HR2 peptide Fc-fusion protein C34-IgG_1_ [45]. To monitor the saturation of the cell surface–expressed Env with sCD4, we utilized the tetrameric CD4-IgG_2_ molecule [46] as a detector (Fig 3A). As sCD4 and CD4-IgG_2_ compete for the same binding site, a decrease in CD4-IgG_2_ staining intensity should reflect the decrease in free CD4 binding sites.

Comparison of the basal epitope exposure curves of the CD4-dependent antibodies revealed a close similarity for the CD4i mAb 17b and V3 crown mAb 1-79. Epitope exposure curves of both antibodies peak at approximately 5 μM sCD4, at which binding of CD4-IgG_2_ is already greatly diminished, indicating a high CD4bs occupancy by sCD4 (Fig 3A). Considering that the coreceptor binding site has substantial overlap with CD4i epitopes and includes the base of the V3 loop [47–49], the peak of the basal epitope exposure curves of 1-79 and 17b indicates the predominance of an Env conformation that is optimally primed for coreceptor interaction, to which we refer hereafter as the optimal CD4i-triggered state. Binding of cluster I mAb 4B3 and of C34-IgG_1_ is not at its maximum at the optimal CD4i-triggered state and increases further upon elevation of sCD4 concentrations (Fig 3A).

We next investigated the epitope exposure for a selection of bnAbs during sCD4 triggering (Fig 3B, S3 and S4 Figs). In line with its known preference for intact closed trimer, the V2 apex bnAb PGT145 showed high binding of CD4-unbound BaL.01 Env but no reactivity with the optimal CD4i-triggered Env. The gp120-gp41 subunit interface bnAb PGT151 and the V3 high-mannose patch bnAb PGT121 also recognized CD4-unbound Env preferentially but maintained substantial binding activity toward the optimal CD4i-triggered state. Whereas the basal epitope exposure curve of bnAb 2G12 (known to interact exclusively with V3 glycans) remained largely unaltered across the probed sCD4 triggering range, the two V3 high-mannose patch bnAbs—PGT128 and PGT135—showed a preference for the optimal CD4i-triggered state. Binding of the two gp41-directed bnAbs, 4E10 (directed against the membrane-proximal external region [MPER]) and VRC34.01 (directed against the FP), was gradually enhanced by increased sCD4 triggering. Thus, similar to MPER mAbs [50,51], VRC34.01 appears to preferentially bind the CD4-bound Env. However, the basal epitope exposure curves of the two bnAbs differ: 4E10 reached its epitope exposure plateau in an sCD4 range close to the optimal CD4i-triggered state, whereas VRC34.01 epitope exposure did not reach a plateau within the tested sCD4 doses. To assess the exposure of gp41 epitopes following CD4 engagement in detail, we probed the binding patterns of a broader panel of gp41-reactive mAbs comprising cluster I (immunogenic loop), cluster II (HR2), and MPER and FP-proximal region (FPPR) mAbs in the on-cell sCD4 triggering assay (S6 Fig). With the exception of MPER mAbs and the reported cluster II mAb 98–6 that showed overlapping patterns, all other gp41 mAbs tested displayed a reactivity pattern identical to the cluster I mAb 4B3 (S6B and S6C Fig, Fig 3A). Though none of the tested gp41 mAbs preferred the CD4-unbound Env conformation, the MPER-targeting bnAbs bound it most efficiently (S6C Fig).

### Resistance to sCD4-induced conformational changes is associated with higher neutralization shielding

**Fig 4:**
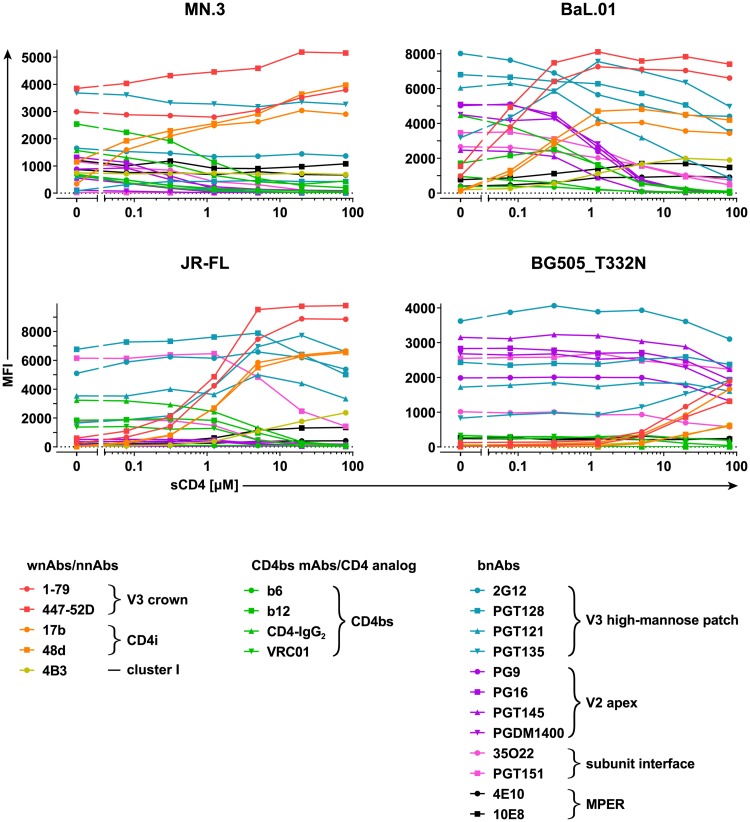
Comparison of the antigenic profiles and CD4-unbound state stability of divergent Envs. On-cell sCD4 triggering of MN.3, BaL.01, JR-FL, and BG505_T332N Envs and assessment of antibody/inhibitor binding at 10 μg/ml with a 20-minute triggering step. MFI staining curves were derived as described in Fig 1. Respective relative MFI staining curves are depicted in S7 Fig and histogram plots of the event distributions in S8 Fig. Data represent a single experiment. BG505_T332N, BG505.W6M.ENV.C2_T332N; CD4bs, CD4 binding site; CD4i, CD4-induced site; Env, envelope glycoprotein; mAb, monoclonal antibody; MFI, mean of fluorescence intensity; MPER, membrane-proximal external region; nnAb, nonneutralizing antibody; sCD4, soluble CD4; V2, second hypervariable; V3, third hypervariable; wnAb, weakly neutralizing antibody.

Next, we probed the effects of sCD4 triggering on virus strains with different levels of general neutralization sensitivity (tiers 1 and 2/3). We included the highly neutralization-sensitive Env MN.3 (subtype B, tier 1A) [52], the relatively neutralization-resistant tier 2 Env JR-FL (subtype B) [53], and the highly neutralization-resistant Env clone BG505.W6M.ENV.C2_T332N (BG505_T332N) (subtype A, tier 2/3) [27,54]. Exposure of epitopes upon sCD4 triggering was assessed at a single antibody concentration (10 μg/ml) that provided a satisfactory signal intensity across all Env/antibody combinations tested. We probed epitope accessibility during gradual sCD4 triggering for three groups of antibodies: (1) weakly neutralizing antibodies (wnAbs) and nonneutralizing antibodies (nnAbs), (2) CD4bs-targeting mAbs, and (3) bnAbs (Fig 4, S7 and S8 Figs).

wnAb/nnAb epitope accessibility in the CD4-unbound stage and the sensitivity to sCD4-triggered conformational changes reflect the known neutralization sensitivity/resistance, open/closed conformation of the four virus strains (Fig 4 and S7A Fig). In particular, binding of wnAbs/nnAbs to MN.3 is already substantial in the CD4-unbound state and further boosted at low sCD4 concentrations. Compared to MN.3, the basal exposure of wnAb/nnAb epitopes on BaL.01 is lower and can be enhanced at relatively modest sCD4 concentrations. In contrast, the accessibility of wnAb/nnAb epitopes on JR-FL is low, and their full exposure requires high concentrations of sCD4. Binding of all wnAbs/nnAbs to BG505_T332N is negligible in the native state, and the sCD4 concentrations necessary to induce their binding are so extreme that we were unable to reach saturation in our assay. Thus, sCD4 doses that suffice to unshield tier 1 and tier 2 Envs failed to fully unmask neutralization-sensitive epitopes on the more neutralization-resistant BG505 trimer.

bnAbs showed similar reactivity patterns in response to sCD4 triggering for all four Envs (Fig 4 and S7B Fig), demonstrating that the conformational preference of bnAbs is well conserved irrespective of the Env genotype and its general neutralization sensitivity/resistance.

The sCD4 triggering assay revealed that the open conformation of MN.3 extends even to gp41 epitopes, as both cluster I and MPER epitopes are already accessible on CD4-unbound Env, and their exposure does not increase upon sCD4 triggering (Fig 4 and S7 Fig). Assessment of epitope exposure by a larger gp41 mAb panel and C34-IgG_1_ (S9 Fig) revealed that the MN.3 trimer assumes a unique, minimally shielded conformation with fully accessible FPPR, cluster I, cluster II, and MPER but effectively concealed FP and HR1 (S9A–S9C Fig). FP and HR1 exposure upon sCD4 triggering closely paralleled the induction of V3 crown and CD4i epitopes on MN.3 (S9D Fig).

### Incomplete neutralization can occur despite high binding capacity to native Env

**Fig 5:**
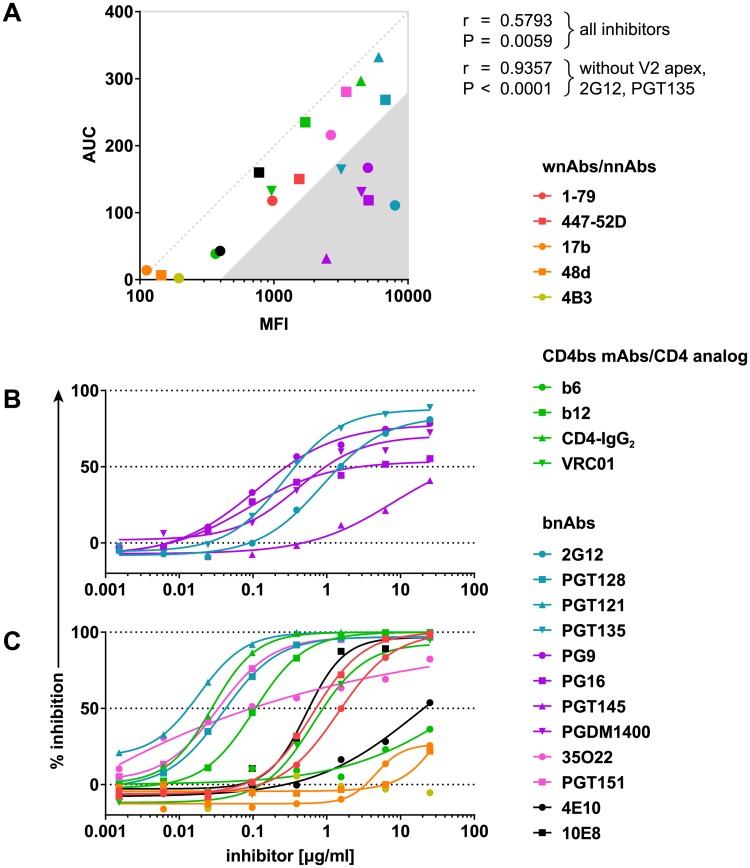
Relationship between BaL.01 neutralization and Env binding capacity. (A) Correlation between the inhibitor binding to the native, CD4-unbound BaL.01 Env (MFI data from Fig 4) and neutralization of the BaL.01 pseudovirus expressed as the AUC derived from neutralization data shown in panels B and C. Pearson’s r coefficients and respective *P* values were calculated taking into consideration either all probed inhibitors or all inhibitors excluding V2 apex bnAbs (PG9, PG16, PGT145, PGDM1400), 2G12, and PGT135. (B, C) Inhibition curves of BaL.01 pseudovirus neutralization assay for (B) V2 apex bnAbs, 2G12, and PGT135 and (C) other mAbs and inhibitors. Binding/neutralization relationships for MN.3, JR-FL, and BG505_T332N Envs as well as the corresponding inhibition curves are depicted in S10 Fig. Data represent a single experiment. AUC, area under the inhibition curve; BG505_T332N, BG505.W6M.ENV.C2_T332N; bnAb, broadly neutralizing antibody; CD4bs, CD4 binding site; Env, envelope glycoprotein; mAb, monoclonal antibody; nnAb, nonneutralizing antibody; MFI, mean of fluorescence intensity; V2, second hypervariable; wnAb, weakly neutralizing antibody.

We next probed the relevance of binding to closed (CD4-unbound) Env conformation for antibody neutralization. A correlation between binding to native BaL.01 Env (mean of fluorescence intensity [MFI] with staining antibody at 10 μg/ml) and neutralization activity (area under the inhibition curve [AUC]) against BaL.01 in the TZM-bl pseudovirus assay signified a wide spectrum of nAbs (Fig 5A). V2 apex bnAbs, 2G12, and to a lesser extent PGT135 differed in this respect, showing comparatively weak neutralization activity despite high binding efficacy. These antibodies exhibited incomplete neutralization of the BaL.01 virus, i.e., an inhibition curve with a top plateau below 100% (Fig 5B and 5C). We observed a similar interrelationship of V2 apex, 2G12, and PGT135 antibody binding and neutralization for MN.3 but not for the more neutralization-resistant viruses JR-FL and BG505_T332N (S10 Fig). The overall positive correlation between binding (MFI) and neutralization activity (AUC) was evident for all four viruses probed irrespective of the open/closed conformation of their native Envs (S10A Fig). In line with the low-level impact of sCD4 triggering on BG505_T332N Env, inhibitor binding in the presence of increasing doses of sCD4 still showed a high correlation with neutralizing activity against the respective virus (Table 1). Notably, this association was rapidly lost for JR-FL and BaL.01, which are more sensitive to sCD4-induced conformational changes (Table 1). The correlation for MN.3 Env was comparable across the entire sCD4 range, highlighting the lack of effective conformational shielding (Table 1).

**Table 1:** Correlation between antibody neutralization and binding to progressively more receptor-triggered forms of the same HIV-1 Env.

	sCD4 [μM]	Env											
		MN.3			BaL.01			JR-FL			BG505_T332N		
		r	*P*		r	*P*		r	*P*		r	*P*	
Pearson correlation	80	0.5364	0.0264	*	−0.1271	0.627	ns	−0.08889	0.7344	ns	0.4389	0.078	ns
	20	0.5236	0.031	*	−0.00437	0.9867	ns	0.04003	0.8788	ns	0.6627	0.0037	**
	5	0.5365	0.0264	*	0.09431	0.7188	ns	0.248	0.3373	ns	0.7374	0.0007	***
	1.25	0.5249	0.0305	*	0.2269	0.3811	ns	0.5837	0.0139	*	0.7524	0.0005	***
	0.3125	0.5619	0.0189	*	0.4478	0.0715	ns	0.7892	0.0002	***	0.745	0.0006	***
	0.078125	0.4939	0.0439	*	0.6129	0.0089	**	0.8114	<0.0001	****	0.7576	0.0004	***
	0	0.505	0.0387	*	0.5987	0.0111	*	0.8398	<0.0001	****	0.7722	0.0003	***

Correlation between MFI of antibody binding to the cell surface–expressed CD4-unbound as well as progressively more CD4-bound MN.3, BaL.01, JR-FL, and BG505_T332N Env and AUC for neutralization of the corresponding pseudovirus. CD4bs-directed inhibitors were excluded from the analysis, as their apparent affinity toward Env in the on-cell sCD4 triggering assay is influenced by direct competition for binding with sCD4. Pearson’s r coefficients and respective two-tailed *P* values are listed. High correlation/low *P* values are shaded in purple; low correlation/high *P* values are shaded in yellow.

Abbreviations: AUC, area under the inhibition curve; BG505_T332N, BG505.W6M.ENV.C2_T332N; CD4bs, CD4 binding site; Env, envelope glycoprotein; MFI, mean of fluorescence intensity; ns, not significant; sCD4, soluble CD4.

* *P* < 0.05.

** *P* < 0.01.

*** *P* < 0.001.

**** *P* < 0.0001.

### HIV-1 bnAbs can differ substantially in their dissociation kinetics

**Fig 6:**
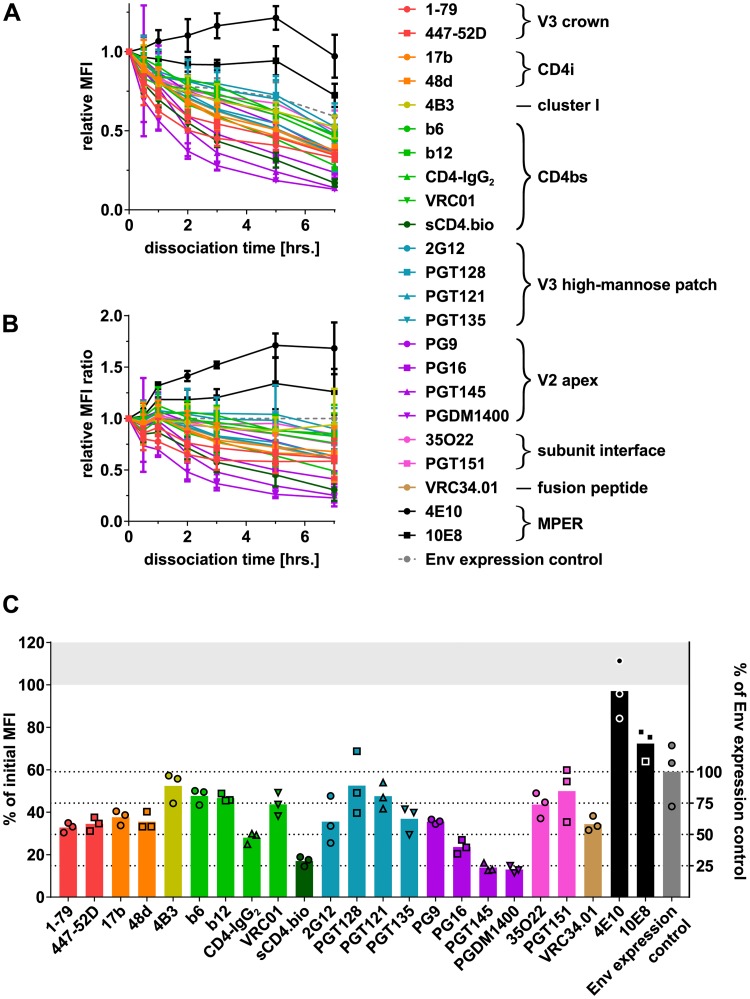
Dissociation of inhibitors from the cell surface–expressed, CD4-unbound BaL.01 SOS Env. BaL.01 SOS Env–expressing HEK 293T cells were stained with 5 μM biotinylated sCD4 or 10 μg/ml of other HIV-1 inhibitors that were then allowed to dissociate at 37 °C until the cells were chemically fixed. The amount of bound inhibitor remaining at the time of fixing was quantified by flow cytometry. Dead cells were excluded from analysis, and background signal from MuLV Env–expressing control cells was subtracted. A separate aliquot of cells stained with 10 μg/ml of 2G12 mAb only after fixing served as an “Env expression control” for the total amount of Env present on the cells at each dissociation time point. (A) MFI values for each inhibitor and Env expression control were normalized to the dissociation time t = 0 (mean [SD] of three independent experiments). (B) The resulting relative MFI values for each inhibitor were divided by the relative MFI value of the Env expression control for the respective time point (mean [SD] of three independent experiments). (C) Relative MFI values for each inhibitor and Env expression control at dissociation time t = 7 hours. Bars indicate the respective means of three replicate values depicted by symbols. Underlying flow cytometry data are depicted in S13 Fig. CD4bs, CD4 binding site; CD4i, CD4-induced site; Env, envelope glycoprotein; HEK, human embryonic kidney; HIV-1, human immunodeficiency virus type 1; mAb, monoclonal antibody; MFI, mean of fluorescence intensity; MPER, membrane-proximal external region; MuLV, murine leukemia virus; sCD4, soluble CD4; V2, second hypervariable; V3, third hypervariable.

A plausible explanation for the disparity in binding and neutralization observed for 2G12 and V2 apex bnAbs would be high dissociation rates of these antibodies [22,55,56]. Alternatively, differences in Env binding and neutralization capacity could also result from the respective assay conditions that may impact antibodies differentially. The on-cell sCD4 triggering assay records binding at room temperature after a relatively short incubation, which may favor the measurement of antibody on-rates. Measurement of neutralization activity involves prolonged incubation, including a preincubation of antibody and virus at 37 °C, creating conditions that can promote antibody dissociation. Prolonged incubation may also irreversibly inactivate trimers (e.g., by arresting in nonfavorable conformations) or cause trimer dissociation accompanied by gp120 shedding [4,21–23]. If present, these effects can impact the readout in both binding and neutralization assays in a virus strain–, antibody-, time-, and temperature-dependent manner. We thus sought to create an assay that enables measurement of antibody dissociation in absence of gp120 shedding. We generated a mutant Env, BaL.01 SOS, which contains a disulfide bond linking gp120-gp41, thereby preventing gp120 dissociation [57]. BaL.01 SOS can be triggered by sCD4 to the same extent as BaL.01 (S11 and S3 Figs). A time-dependent loss in gp120 antibody signal was observed for BaL.01 wild type, which is in line with the ability of gp120 to dissociate from BaL.01 but, as expected, not for BaL.01 SOS (S11 Fig and Fig 1). As BaL.01 SOS expression was higher, BaL.01 SOS/BaL.01 binding ratios were normalized for expression based on antibody 2G12 and the reactivity of both Envs with a large panel of antibodies compared (S12 Fig). The cell surface–expressed wild-type BaL.01 and BaL.01 SOS proved antigenically very similar. We only observed localized differences in antibody binding to BaL.01 SOS compared to BaL.01 wild type that centered on CD4i and gp41 epitopes. CD4bs, V3 high-mannose patch, and V2 apex and subunit interface reactivity was comparable (S12C Fig). Probing the dissociation of a panel of mAbs, CD4-IgG_2_, and sCD4 after binding to BaL.01 SOS Env–expressing cells at 37 °C (Fig 6 and S13 Fig), we observed the highest dissociation rates for V2 apex mAbs, PGT135, and 2G12 (Fig 6A–6C). In contrast, the signal for MPER bnAbs decreased very slowly or even increased slightly over time (Fig 6A and 6B). Antibody dissociation curves for wild-type BaL.01 closely resembled the patterns observed for the SOS mutant with the expected exception of a rapid decline following sCD4 or CD4-IgG_2_ treatment known to induce gp120 shedding (S14 Fig). The fact that we observed equivalent dissociation patterns of antibodies on both BaL.01 wild type and BaL.01 SOS confirms that the decrease in binding we observe for certain antibodies is not due to shedding but indeed reflects high dissociation rates.

### Structural antagonism with CD4-triggered Env promotes incomplete neutralization by V2 apex bnAbs

**Fig 7:**
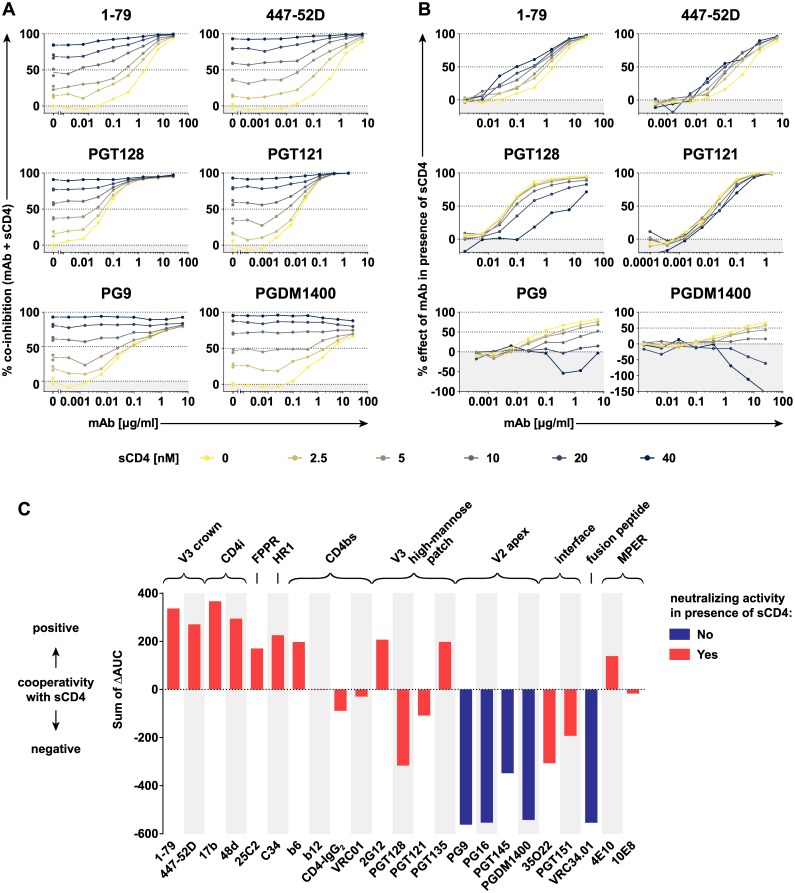
Neutralization cooperativity between sCD4 and different HIV-1 nAbs/inhibitors. (A) Neutralizing activity of antibodies against BaL.01 pseudovirus was assayed in the presence of increasing sCD4 concentrations (0–40 μM). Data represent a single experiment. See S15 Fig for coinhibition curves of further nAbs/inhibitors with sCD4. (B) The percent effect of a nAb in the presence of sCD4 was calculated relative to the residual infectivity measured with sCD4 at the respective concentration in the absence of nAb/inhibitor. See S16 Fig for percent effect curves of further nAbs/inhibitors. (C) The difference between the area under the percent effect curve of a nAb/inhibitor in the presence of 2.5, 5, 10, 20, or 40 μM sCD4 and the area under its percent effect curve in sCD4 absence (i.e., its neutralization curve) was calculated and designated as ΔAUC. The neutralization cooperativity of a particular nAb/inhibitor was then calculated as the sum of the five ΔAUC values. The color code of the bars denotes if nAbs/inhibitors retained (red) or lost (blue) neutralizing activity at the highest nAb/inhibitor and sCD4 dose probed (see Fig 7B and S16 Fig). AUC, area under the inhibition curve; CD4i, CD4-induced site; FPPR, fusion peptide–proximal region; HIV-1, human immunodeficiency virus type 1; HR1, heptad repeat 1; mAb, monoclonal antibody; MPER, membrane-proximal external region; nAb, neutralizing antibody; sCD4, soluble CD4; V2, second hypervariable; V3, third hypervariable.

Neutralization of HIV-1 before CD4 attachment is considered critical, as access of antibodies to the CD4-engaged Env on cells may be more constrained, and rapid progression toward fusion likely limits possibilities of antibody interference [58]. However, antibodies that retain post-CD4-attachment activity may increase their window of opportunity and also potentially neutralize the virus better in the setting of cell–cell transmission [18,59]. It is thus of high interest to determine if and which bnAbs have a post-CD4-attachment activity. CD4 triggering has been shown to have a negative effect on the binding of V2 apex bnAbs PG9 and PG16 [60], but neutralization capacity of these and other bnAbs in presence of CD4 has thus far not been systematically investigated. Probing neutralization of BaL.01 by antibodies and other Env-directed entry inhibitors in the presence of increasing doses of sCD4 (Fig 7A and S15 Fig), we observed diverse patterns of both positive and negative neutralization cooperativity (Fig 7B and 7C, S16 Fig). To assess if bnAbs retain neutralization activity post CD4 triggering, we calculated the antibody-contributed inhibition in the coinhibition with sCD4 (Fig 7B and S16 Fig). When an antibody exhibited no additional inhibitory effect at the highest antibody and sCD4 dose tested, we recorded this as loss of antibody-mediated neutralization (Fig 7C). Antibodies with positive CD4 cooperativity gained in neutralizing activity. These included antibodies directed to the V3 crown, the CD4i and HR2 peptides, in line with previous findings [61–67]. CD4bs bnAbs showed no cooperativity with CD4, consistent with their shared binding site with sCD4. Cooperativity of sCD4 with MPER bnAbs was positive but small (4E10) or negligible (10E8). The effect of CD4 triggering differed for V3 high-mannose patch bnAbs. PGT135 and 2G12 showed a positive cooperativity with a slightly improved neutralization capacity in presence of CD4. In contrast, PGT121 and PGT128 displayed an intermediate negative cooperativity with CD4. Both showed reduced capacity to neutralize post-CD4 engagement but retained considerable neutralization activity. bnAbs with strong negative CD4 cooperativity completely lost neutralization activity. The FP-directed bnAb VRC34.01 and all probed V2 apex bNAbs fell into this category. Collectively, this suggests that the high off-rate of V2 bnAbs in binding to the native Env and their incapacity to interact with CD4-triggered Env are reflected in the incomplete neutralization commonly observed for these bnAbs.

## Discussion

Definition of the various conformations the HIV-1 Env trimer can adopt is one of the main gaps in the knowledge of the HIV-1 entry process and its neutralization [68,69]. Most approaches toward this are technically complex and not easily scalable [31,33,34,41,42,70,71]. Here, we provide information on the antigenic landscape of multiple distinct receptor-triggered Env forms with varying CD4bs occupancy that is based on a comparatively simple binding assay setup. Our study builds on a tremendous body of work dedicated to unraveling the interactions of the Env trimer with CD4 and the conformational stages the trimer adopts upon CD4 engagement [43–45,72–74]. To be able to explore Env in a close to native setting, we assessed cell surface–expressed Env trimers by flow cytometry, a widely used setup [6–8,51,60,75–77]. A fine-tuned composition of the assay—which controls for a range of factors including temporal kinetics of CD4 triggering, allosteric effects of antibodies, and Env inactivation through gp120 shedding—allowed us to trace and systematically investigate the varying conformations of native and CD4-bound Env. Increasing the average occupancy of CD4bs on the trimers manipulates the equilibrium of Env conformational states. The successive CD4-triggered conformations that are generated at different sCD4 concentrations represent the majority species at the given assay condition. As both unliganded and CD4-bound states are metastable, our assay is best viewed as providing snapshots of a shifting equilibrium between conformational states.

By exposing the Env to a wide range of sCD4 concentrations, we demonstrate a simple strategy to promote successive receptor-induced Env states to form, allowing their detailed antigenic characterization. What advances our setup over any described thus far is the establishment of individual basal epitope exposure curves for each antibody tested. With this, we minimize the allosteric effects of antibody binding on Env that might obscure the true conformational preference of antibodies. Our study shows how this limitation can be overcome and how a simple Env binding setup can be turned into a powerful antigenic characterization tool.

Whereas our study focused solely on the effects of CD4 engagement on antibody epitope exposure, extending these analyses in future studies to evaluate the allosteric effects of antibody binding on the trimer and to explore potential multistep binding of antibodies to the trimer will be of high interest. In the present study, we sought to limit the allosteric effects of antibodies by carefully titrating antibody doses and restricting the readout to low doses. Differential epitope exposure at high and low antibody doses that we note in these dose-finding experiments (S3 Fig) is an indication of antibody-induced allosteric effects at higher antibody doses, highlighting potential avenues for further investigations.

Our results complement recent investigations on the dynamic conformational rearrangements of the HIV-1 trimer [5,41]. The on-cell sCD4-triggering assay allows for fine antigenic mapping of the conformational dynamics of the full-length Env trimer and to resolve dynamic changes in the antigenic landscape upon receptor triggering. By assessing the response of Env trimers to sCD4, triggering the assay further delivers a measure of trimer stability of the CD4-unbound state by disclosing how refractory a respective trimer to receptor-induced conformational changes is. This recommends our assay for screening of stable Env trimers considered for immunogen development.

Binding efficacy of nAbs to native HIV-1 Env is a known predictor of in vitro neutralization potency [4,15,16,78]. Beyond confirming the strong link between neutralization and trimer binding [4,15,16,78], our analyses provide the first survey, to our knowledge, of these relationships that covers the probed Env from the native state through the full range of CD4-triggered conformations. Our results further expand on prior findings by highlighting that exceptions exist. Certain antibodies can bind the unliganded Env with high affinity but fail to completely neutralize the corresponding virus as shown for the V2 apex bnAbs and 2G12 bnAb (Fig 5). A high rate of antibody dissociation can result in reversible neutralization as previously shown for 2G12 [22,56], and a high off-rate of bnAb binding has been suggested to result in incomplete neutralization [55]. Here, we observe incomplete neutralization occurring predominantly for antibodies that display a high dissociation rate. Incomplete neutralization of HIV-1 is a commonly noted phenomenon in different assay systems, and other factors such as differential glycosylation and conformational heterogeneity of Env can affect its appearance [79–82]. In the present study, we show that the high off-rate in binding to the native Env and incapacity to recognize and neutralize CD4-triggered Env are two factors that may contribute strongly to the incomplete neutralization by V2 bnAbs.

A key finding of our study is that we show several bnAbs to preferentially bind the CD4-triggered Env. This may indicate a capacity of these antibodies to neutralize both pre- and post-CD4 engagement, which needs to be explored. Inferring neutralization capacity post CD4 triggering directly from binding activity alone is, however, not straightforward. Accessibility of free virus and cell-bound virus may differ. Certain Env conformations that allow high epitope accessibility may be sampled beyond a relevant step in entry that can be blocked. In line with this, we observed an intriguing disparity for bnAb VRC34.01, which showed enhanced binding upon CD4 triggering but decreased neutralization activity that will be interesting to tease apart in forthcoming studies. Overall, we found neutralization and binding activity to correlate well for native and partially triggered trimer but not for the fully opened trimer (Table 1). A strong correlation with native trimer fits with the notion that inhibition prior to receptor engagement is important [4,15,16,78]. Adding to this, our data suggest that neutralization in early stages of CD4 triggering may be possible for certain types of antibodies.

Current assay systems predominantly record pre-CD4-attachment neutralization effects of antibodies [83]. Preincubation of antibodies and virions before addition to target cells favors the pre-CD4-attachment activity. Proof of post-CD4-attachment neutralization activity has long been established for MPER bnAbs but remained less studied for gp120 antibodies [22,59]. In light of our new findings, post-CD4-attachment activity should be systematically investigated to understand if our current screening systems capture this activity properly and to define its role in neutralization in vivo. Retaining neutralization activity beyond CD4 triggering may be key when antibody binding is reversible. Likewise, the capacity to neutralize post-CD4 engagement has been implicated in blocking cell–cell transmission of HIV-1 where engagement of CD4 is more rapid than in the setting of free virus infection [18,59].

Collectively, the findings we made using the on-cell sCD4 triggering assay contribute to a refined view of the process of HIV-1 entry and its inhibition. Our study highlights a continued need to resolve which Env conformations are neutralization-relevant. This will not only be interesting from a mechanistic point of view but will also provide guidance for immunogen development.

## Material and methods

### Cell lines, inhibitors, and plasmids

HEK 293T cells were obtained from the American Type Culture Collection and TZM-bl cells [84] through the NIH AIDS Reagent Program, Division of AIDS, NIAID, NIH. Cell lines were maintained in DMEM, high glucose, pyruvate (Gibco, Thermo Fisher Scientific, Waltham, MA, USA) supplemented with 10% heat-inactivated FBS (Gibco, Thermo Fisher Scientific, Waltham, MA, USA), 100 U/ml penicillin, and 100 μg/ml streptomycin (Gibco, Thermo Fisher Scientific, Waltham, MA, USA) at 37 °C, 5% CO_2_, and 80% relative humidity. The sources of inhibitors and plasmids used in this study are listed in S1 and S2 Tables, respectively.

### Production of recombinant two-domain sCD4 (sCD4-183_avi_his.bio)

A codon-optimized sequence corresponding to 183 N-terminal amino acid residues of mature human CD4 followed by AviTag (Avidity, Aurora, CO, USA), GSG linker, and 8xHis-tag was cloned into the pET-32a(+) (Merck KGaA, Darmstadt, Germany) expression vector so as to contain only one additional N-terminal methionine residue. The resulting plasmid was cotransformed together with the pBirAcm biotin ligase expression plasmid (Avidity, Aurora, CO, USA) into SHuffle T7 Express Competent *Escherichia coli* (New England Biolabs, Ipswich, MA, USA). Bacteria were kept under antibiotic selection pressure in TYH medium (2% w/v tryptone, 1% w/v yeast extract, 1.1% w/v HEPES, 0.5% w/v NaCl, 0.1% [w/v] MgSO_4_ [pH = 7.3]) to maintain both plasmids. Bacterial cultures, of which the optical density at 600 nm reached 0.7–0.8, were induced with 50 μM Isopropyl β-D-1-thiogalactopyranoside in the presence of 50 μM D-biotin for 20 hours at 16 °C. Cells were mechanically disrupted in extraction buffer (50 mM phosphate, 250 mM NaCl, 20 mM imidazole, 20% v/v glycerol, 0.2% TWEEN-20 [pH = 7.4]). The recombinant protein in the soluble fraction was bound to Ni-NTA Superflow resin (Qiagen, Venlo, the Netherlands). After washing with 60 resin volumes of wash buffer 1 (50 mM phosphate, 250 mM NaCl, 20 mM imidazole, 20% v/v glycerol [pH = 7.4]) and 20 resin volumes of wash buffer 2 (50 mM phosphate, 20 mM imidazole, 10% v/v glycerol [pH = 7.4]), the protein was eluted with 8 resin volumes of elution buffer (50 mM phosphate, 250 mM imidazole, 10% v/v glycerol [pH = 7.4]). Finally, the protein was purified by size-exclusion chromatography in FPLC buffer (50 mM phosphate, 10% v/v glycerol [pH = 7.4]) on a HiLoad 26/600 Superdex 200 column/Äktaprime plus FPLC system (GE Healthcare, Uppsala, Sweden).

### Flow cytometric analysis of antibody binding to cell surface–expressed Env

A total of 1.25 × 10^5^ HEK 293T cells per well were seeded in 1 ml of culture medium in 12-well tissue culture plates and incubated at 37 °C. Twenty-four hours later, cells in each well were transfected with a total of 1 μg DNA (Env expression plasmid and pCMV-*rev* expression helper plasmid in 4:1 ratio) mixed with 3 μg 25-kDa linear PEI or 40-kDa PEI MAX (Polysciences, Warrington, PA, USA) in 200 μl 150-mM NaCl. After settling the DNA-PEI complexes by a short spin (3 minutes, room temperature, 300*g*), the cells were incubated for 36 hours at 37 °C. All subsequent steps were carried out at room temperature. Cells were harvested, pooled, and distributed into 96-well round-bottom tissue culture plates for the individual staining reactions. For each staining reaction, cells were washed once with 200 μl staining buffer (DPBS [Gibco, Thermo Fisher Scientific, Waltham, MA, USA] with 2% heat-inactivated FBS [Gibco, Thermo Fisher Scientific, Waltham, MA, USA], and 2 mM EDTA) and stained for 20 minutes (unless indicated otherwise) in 20 μl of staining buffer with 10 μg/ml of primary antibody (unless indicated otherwise) with or without the presence of sCD4. After washing twice with 200 μl staining buffer, a secondary staining mix of 30 μl of staining buffer with 1:1,000 diluted APC-conjugated F(ab')₂ fragment goat anti-human IgG (Jackson ImmunoResearch, West Grove, PA, USA) or APC-conjugated streptavidin (BioLegend, San Diego, CA, USA) was added to the cells for 20 minutes. Following two washes with staining buffer, the cells were resuspended in 100 μl staining buffer with 0.1 μg/ml propidium iodide (BD Biosciences, San Jose, CA, USA). Flow cytometry data were acquired on the FACSVerse system (BD Biosciences, San Jose, CA, USA) and analyzed using FlowJo 10 software (FlowJo, Ashland, OR, USA). Arithmetic mean of APC fluorescence intensity (MFI) was calculated for the gated propidium iodide–negative single-cell population as a measure of primary antibody binding to the live cells in each staining reaction.

For the experiments that required cell fixation, the protocol was conducted with the following modifications: A four times greater number of cells was used per staining reaction. Instead of propidium iodide in the final resuspension buffer, 1:1,000 Zombie Green Fixable Viability Dye (BioLegend, San Diego, CA, USA) was included in the primary staining mix of 30 μl total volume. After incubation with the primary staining mix, the cells were washed once with 200 μl DPBS, a further 200 μl of DPBS were added, and cells were put to 37 °C. At a specified time point, the DPBS was replaced with a fixing solution of 3% paraformaldehyde in DPBS. After 20-minute incubation at room temperature, the cells were washed twice with DPBS before adding the secondary staining mix. For postfixation cell staining, a simultaneous primary/secondary staining step was used with APC-conjugated F(ab')₂ fragment goat anti-human IgG (Jackson ImmunoResearch, West Grove, PA, USA) diluted 1:1,000 in staining buffer together with the primary mAb 2G12 at 10 μg/ml.

In the on-cell sCD4 triggering assay, the MFI values for cells subjected to the same experimental conditions other than a different concentration of sCD4 were regarded as one data series, the MFI staining curve. To obtain a relative MFI staining curve, each of the MFI values within an MFI staining curve was divided by the maximum MFI value present within the same MFI staining curve. In the inhibitor dissociation assay, the relative MFI values for each inhibitor/control were calculated by dividing the MFI values for each time point by the MFI value for the time point t = 0. In the on-cell sCD4 triggering assay, the basal epitope exposure curve was selected from the relative MFI staining curves of each individual antibody/inhibitor based on the following criteria: lowest concentration of the tested antibody/inhibitor that yields at least a 10-fold higher MFI signal over MuLV background at the curve maximum.

We used CD4-IgG_2_ as an indicator for CD4bs saturation to set an upper bound on the CD4 occupancy in the assay. CD4-IgG_2_ has a higher affinity compared to sCD4 because of the capacity of multivalent binding. It further allows detection via the Fc. Employing CD4-IgG_2_ as a reference point allowed us to define if and which CD4-triggered Env stages occur below saturating conditions.

### Production of Env-pseudotyped HIV-1 particles

A total of 2.25 × 10^6^ HEK 293T cells were seeded in 20 ml of culture medium in a T75 flask and incubated at 37 °C. Twenty-four hours later, cells were transfected with a total of 20 μg DNA (Env expression plasmid and pNL-lucAM HIV-1 backbone plasmid in 1:3 ratio) mixed with 60 μg 25-kDa linear PEI (Polysciences, Warrington, PA, USA) in 4 ml 150-mM NaCl. At 6–18 hours post transfection, the culture medium was exchanged. Culture supernatant was harvested 48 hours post transfection, vacuum filtered through a 0.22-μm-pore-size membrane, and frozen as pseudovirus stock.

### Neutralization assay

The TZM-bl based pseudovirus neutralization assay was conducted essentially as previously described [85]. TZM-bl cells (1 × 10^4^) in 100 μl of culture medium containing 20 μg/ml DEAE-Dextran (Amersham Biosciences, Uppsala, Sweden) were seeded in each well of white, 96-well, clear flat-bottom tissue culture plates (Greiner Bio-One, Kremsmünster, Austria) and incubated at 37 °C. Twenty-four hours later, pseudovirus was preincubated with serially diluted Env-directed inhibitors in culture medium for 1 hour at 37 °C or alternatively 20 minutes at room temperature for sCD4 coinhibition experiments. The pseudovirus-inhibitor mixture (100 μl) was added to the TZM-bl cells. Luciferase reporter gene expression was assessed 48 hours post infection with Bright-Glo Luciferase Assay System (Promega, Fitchburg, WI, USA) on the Dynex MLX luminometer (Dynex Technologies, Chantilly, VA, USA). Virus input was chosen to yield virus infectivity corresponding to 10,000–40,000 relative light units (RLU) on the medium sensitivity setting in the absence of inhibitors. Prism 7 software (GraphPad Software, La Jolla, CA, USA) was used to fit 4-parameter logistic curves to the data and to calculate the area under each neutralization curve (AUC).

To quantify the neutralization cooperativity of a nAb/inhibitor with sCD4, the percent effect of a nAb in the presence of sCD4 was calculated relative to the residual infectivity measured with sCD4 at the respective concentration in the absence of nAb/inhibitor. The difference between the area under the percent effect curve of a nAb/inhibitor in the presence of 2.5, 5, 10, 20, or 40 μM sCD4 and the area under its percent effect curve in sCD4 absence (i.e., its neutralization curve) was calculated and designated as ΔAUC. The neutralization cooperativity of a particular nAb/inhibitor was then calculated as the sum of the five ΔAUC values.

### Statistical analysis

Pearson’s r coefficients and respective *P* values were calculated using Prism 7 software (GraphPad Software, La Jolla, CA, USA).

### Structure figure preparation

The structure figure was prepared with PyMOL Molecular Graphics System 1.7 software (Schrödinger, New York, NY, USA) using the referenced protein structure database file.

## Supporting information

S1 Fig Histogram plots of on-cell sCD4 triggering with BaL.01 Env and 1-79 mAb. Data related to Fig 1. HEK 293T cells expressing the HIV-1 BaL.01 Env were stained with the indicated concentrations of 1-79 mAb in the presence of increasing sCD4 concentrations for 10 or 60 minutes. Inhibitor binding to MuLV Env–expressing cells in the absence of sCD4 is shown as a measure of nonspecific cell surface staining. Only the fluorescence intensities of live cells are displayed. Env, envelope glycoprotein; HEK, human embryonic kidney; HIV-1, human immunodeficiency virus type 1; mAb, monoclonal antibody; MuLV, murine leukemia virus; sCD4, soluble CD4.
(PDF)

S2 Fig Histogram plots of time-resolved on-cell sCD4 triggering assays with BaL.01 Env. Data related to Fig 2. HEK 293T cells expressing the HIV-1 BaL.01 Env were stained with 0.1 μg/ml of 1-79 or PGT145 mAb in the presence of increasing sCD4 concentrations for up to 21 minutes. Inhibitor binding to MuLV Env–expressing cells in the absence of sCD4 is shown as a measure of nonspecific cell surface staining. Only the fluorescence intensities of live cells are displayed. Env, envelope glycoprotein; HEK, human embryonic kidney; HIV-1, human immunodeficiency virus type 1; mAb, monoclonal antibody; MuLV, murine leukemia virus; sCD4, soluble CD4.
(PDF)

S3 Fig Determining the basal epitope exposure curves of Env-directed inhibitors on BaL.01 Env. Data related to Fig 3 and S4 Fig. Env-directed inhibitors were titrated in the on-cell sCD4 triggering assay with a 20-minute triggering step. MFI staining curves and relative MFI staining curves were derived as described in Fig 1. MFI staining curves with less than 10-fold higher signal over MuLV background at the peak and the corresponding normalized MFI staining curves are plotted as dashed curves. Data represent a single experiment. Env, envelope glycoprotein; MFI, mean of fluorescence intensity; MuLV, murine leukemia virus; sCD4, soluble CD4.
(PDF)

S4 Fig Histogram plots of on-cell sCD4 triggering assays with BaL.01 Env and various Env-directed inhibitors. Data related to Fig 3 and S3 Fig. HEK 293T cells expressing the HIV-1 BaL.01 Env were stained with various Env-directed inhibitors at the indicated concentrations in the presence of increasing concentrations of sCD4 for 20 minutes. Inhibitor binding to MuLV Env–expressing cells in the absence of sCD4 is shown as a measure of nonspecific cell surface staining. Only the fluorescence intensities of live cells are displayed. Env, envelope glycoprotein; HEK, human embryonic kidney; HIV-1, human immunodeficiency virus type 1; MuLV, murine leukemia virus; sCD4, soluble CD4.
(PDF)

S5 Fig Reproducibility of the on-cell sCD4 triggering assay. HEK 293T cells expressing the HIV-1 BaL.01 Env were stained with the indicated concentrations of the CD4i mAb 48d in the presence of increasing sCD4 concentrations for 20 minutes. (A) MFI staining curves and relative MFI staining curves were derived as described in Fig 1. (B) Histogram plots corresponding to data shown in panel A. Inhibitor binding to MuLV Env–expressing cells in the absence of sCD4 is shown as a measure of nonspecific cell surface staining. Only the fluorescence intensities of live cells are displayed. The experiments were performed on two different days using different cell batches. CD4i, CD4-induced site; Env, envelope glycoprotein; HEK, human embryonic kidney; HIV-1, human immunodeficiency virus type 1; mAb, monoclonal antibody; MFI, mean of fluorescence intensity; MuLV, murine leukemia virus; sCD4, soluble CD4.
(PDF)

S6 Fig sCD4-triggered exposure of antibody epitopes on the gp41 subunit of cell surface–expressed BaL.01 Env. (A) Domain organization of HIV-1 gp41 with locations of major antibody epitope clusters (HXB2 numbering). Depicted is a crystal structure of stabilized soluble X1193.c1 Env trimer (PDB ID 5FYJ). All three gp120 subunits are in gray; three gp41 subunits are in either black, dark gray, or rainbow (blue to red). On-cell sCD4 triggering of BaL.01 Env was carried out with inhibitors at a fixed concentration of 10 μg/ml with a 20-minute triggering step. MFI (B) and relative MFI (C) staining curves of gp41-directed mAbs in BaL.01 on-cell sCD4 triggering assay were obtained as described in Fig 1. (D) Histogram plots corresponding to data shown in panels B and C. Inhibitor binding to MuLV Env–expressing cells in the absence of sCD4 is shown as a measure of nonspecific cell surface staining. Only the fluorescence intensities of live cells are displayed. Data represent a single experiment. CT, cytoplasmic tail; Env, envelope glycoprotein; HIV-1, human immunodeficiency virus type 1; mAb, monoclonal antibody; MFI, mean of fluorescence intensity; MuLV, murine leukemia virus; PDB, Protein Data Bank; sCD4, soluble CD4; TM, transmembrane region.
(PDF)

S7 Fig Triggering conformational changes on cell surface–expressed HIV-1 Envs of different origin and general neutralization resistance. Related to Fig 4 and S8 Fig. MFI staining curves of selected gp120- and gp41-directed inhibitors from the on-cell sCD4 triggering assay with HIV-1 MN.3, BaL.01, JR-FL, and BG505_T332N Envs depicted in Fig 4 were normalized to their maxima. For clarity, the resulting relative MFI staining curves are shown separately for wnAbs/nnAbs and CD4bs-directed reagents (A) and for bnAbs (B). BG505_T332N, BG505.W6M.ENV.C2_T332N; bnAb, broadly neutralizing antibody; CD4bs, CD4 binding site; Env, envelope glycoprotein; HIV-1, human immunodeficiency virus type 1; MFI, mean of fluorescence intensity; nnAb, nonneutralizing antibody; sCD4, soluble CD4; wnAb, weakly neutralizing antibody.
(PDF)

S8 Fig Histogram plots of on-cell sCD4 triggering assays of different HIV-1 Envs. Related to Fig 4 and S7 Fig. Binding of a panel of HIV-1 Env–directed inhibitors at 10 μg/ml in the presence of increasing sCD4 concentrations to HEK 293T cells expressing the HIV-1 MN.3, BaL.01, JR-FL, and BG505_T332N Envs. Inhibitor binding to MuLV Env–expressing cells in the absence of sCD4 is shown as a measure of nonspecific cell surface staining. Only the fluorescence intensities of live cells are displayed. BG505_T332N, BG505.W6M.ENV.C2_T332N; Env, envelope glycoprotein; HEK, human embryonic kidney; HIV-1, human immunodeficiency virus type 1; MuLV, murine leukemia virus; sCD4, soluble CD4.
(PDF)

S9 Fig Tracking sCD4-induced conformational changes of MN.3 Env with C34-IgG_1_. (A, B) MFI staining curves of selected gp120- and gp41-directed inhibitors from on-cell sCD4 triggering of MN.3 Env were obtained as in Fig 4. MFI staining curves are shown for all (A) or only gp41-directed inhibitors (B) for clarity. (C, D) MFI staining curves normalized to their maxima (relative MFI staining curves) are shown for gp41-targeting mAbs (C) and selected gp120- and gp41-directed inhibitors (D). (E) Underlying flow cytometry data in the form of histogram plots. Inhibitor binding to MuLV Env–expressing cells in the absence of sCD4 is shown as a measure of nonspecific cell surface staining. Only the fluorescence intensities of live cells are displayed. Data represent a single experiment. Env, envelope glycoprotein; mAb, monoclonal antibody; MFI, mean of fluorescence intensity; MuLV, murine leukemia virus; sCD4, soluble CD4.
(PDF)

S10 Fig Relationship between inhibitor binding to cell surface–expressed HIV-1 Env and neutralization of a virus pseudotyped with the corresponding Env. Related to Fig 5. (A) Correlation between the MFI of inhibitor binding to the native, CD4-unbound MN.3, BaL.01, JR-FL, and BG505_T332N Env (MFI data from Fig 4) and the AUC derived from neutralization data of the corresponding pseudovirus shown in panel B. Pearson’s r coefficients and respective *P* values were calculated taking into consideration either all probed inhibitors or all inhibitors excluding V2 apex bnAbs (PG9, PG16, PGT145, PGDM1400), 2G12, and PGT135. (B) Neutralization assay of HIV-1 pseudovirus carrying MN.3, BaL.01, JR-FL, and BG505_T332N Envs with multiple cases of incomplete neutralization. Data represent a single experiment. AUC, area under the inhibition curve; BG505_T332N, BG505.W6M.ENV.C2_T332N; bnAb, broadly neutralizing antibody; Env, HIV-1 envelope glycoprotein; HIV-1, human immunodeficiency virus type 1; MFI, mean of fluorescence intensity; MuLV, murine leukemia virus; sCD4, soluble CD4; V2, second hypervariable.
(PDF)

S11 Fig Triggering conformational changes in cell surface–expressed BaL.01 SOS Env by sCD4. HEK 293T cells expressing the HIV-1 BaL.01 SOS Env were stained with the indicated concentrations of the CD4i mAb 17b in the presence of increasing sCD4 concentrations for 10 minutes (A, B) or 60 minutes (C, D). MFI staining curves (A, C) and relative MFI staining curves (B, D) were derived as described in Fig 1. (E) Histogram plots corresponding to data shown in panels A–D. Inhibitor binding to MuLV Env–expressing cells in the absence of sCD4 is shown as a measure of nonspecific cell surface staining. Only the fluorescence intensities of live cells are displayed. Data represent a single experiment. CD4i, CD4-induced site; Env, envelope glycoprotein; HEK, human embryonic kidney; HIV-1, human immunodeficiency virus type 1; mAb, monoclonal antibody; MFI, mean of fluorescence intensity; MuLV, murine leukemia virus; sCD4, soluble CD4.
(PDF)

S12 Fig Comparison of the antigenic profiles of cell surface–expressed BaL.01 wild-type and BaL.01 SOS Envs. HEK 293T cells expressing the MuLV, HIV-1 BaL.01, or HIV-1 BaL.01 SOS Env were stained with 5 μg/ml of the indicated HIV-1 Env–directed inhibitors. The amount of antibody bound to the cell surface was quantified by flow cytometry. Dead cells were excluded from analysis, and background signal from the MuLV Env–expressing control cells was subtracted from the signal of the HIV-1 Env–expressing cells. The resulting MFI values are depicted in panel A and the derived BaL.01 SOS/wild-type MFI ratios in panel B. To correct for general difference in expression between the two Envs and to allow for an easier visual readout of binding differences, the MFI ratios from panel B were normalized to the MFI ratio of mAb 2G12 (C). Env, envelope glycoprotein; HEK, human embryonic kidney; HIV-1, human immunodeficiency virus type 1; mAb, monoclonal antibody; MFI, mean of fluorescence intensity; MuLV, murine leukemia virus.
(PDF)

S13 Fig Histogram plots of flow cytometry data from inhibitor dissociation assay with BaL.01 SOS Env. Related to Fig 6. BaL.01 SOS Env–expressing HEK 293T cells were stained with 5 μM biotinylated sCD4 or 10 μg/ml of other HIV-1 inhibitors that were then allowed to dissociate at 37 °C until the cells were chemically fixed at the indicated time points. Cells that were stained with 10 μg/ml of 2G12 mAb only after fixing served as an “Env expression control” to depict the changing amount of Env on the cell surface during the experiment. (A, B, C) Histogram plots from three independent experiments. Inhibitor binding to MuLV Env–expressing cells at t = 0 is shown as a measure of nonspecific cell surface staining. Only the fluorescence intensities of live cells are displayed. Env, envelope glycoprotein; HEK, human embryonic kidney; HIV-1, human immunodeficiency virus type 1; mAb, monoclonal antibody; MFI, mean of fluorescence intensity; MuLV, murine leukemia virus; sCD4, soluble CD4.
(PDF)

S14 Fig Dissociation of inhibitors from the cell surface–expressed, CD4-unbound BaL.01 Env. Data from the dissociation assay were acquired and processed as described in Fig 6. (A) MFI values for each inhibitor and Env expression control were normalized to the dissociation time t = 0. (B) The resulting relative MFI values for each inhibitor were divided by the relative MFI value of the Env expression control for the respective time point. (C) Relative MFI values for each inhibitor and Env expression control at dissociation time t = 7 hours. (D) Underlying flow cytometry data in the form of histogram plots. Inhibitor binding to MuLV Env–expressing cells at t = 0 is shown as a measure of nonspecific cell surface staining. Only the fluorescence intensities of live cells are displayed. Data represent a single experiment. Env, envelope glycoprotein; MFI, mean of fluorescence intensity; MuLV, murine leukemia virus.
(PDF)

S15 Fig Raw coinhibition curves of different Env-directed inhibitors with sCD4. Related to Fig 7 and S16 Fig. Neutralization potency of different Env-directed inhibitors against BaL.01 pseudovirus was assayed in the presence of increasing sCD4 concentrations. The percent coinhibition value was set relative to the infectivity measured with no inhibitor present. Data depict a single experiment or a representative experiment of two to three conducted. Env, envelope glycoprotein; sCD4, soluble CD4.
(PDF)

S16 Fig Synergy of BaL.01 virus inhibition by different Env-directed inhibitors and sCD4. Related to Fig 7 and S15 Fig. Neutralization potency of different Env-directed inhibitors against BaL.01 pseudovirus was assayed in the presence of increasing sCD4 concentrations. The percent effect value of an inhibitor was set relative to the infectivity measured with sCD4 at the specified concentration in the inhibitor absence. Data depict a single experiment or a representative experiment of two to three conducted. Env, envelope glycoprotein; sCD4, soluble CD4.
(PDF)

S1 Table Origin and specificity of HIV-1 Env–directed inhibitors. Env, envelope glycoprotein.
(PDF)

S2 Table Origin of plasmids. (PDF)

S1 Data Excel spreadsheet containing, in separate sheets, the underlying numerical data used in all figures. (XLSX)

## Abbreviations

AUC: area under the inhibition curve
BG505_T332N: BG505.W6M.ENV.C2_T332N
bnAb: broadly neutralizing antibody
CD4bs: CD4 binding site
CD4i: CD4-induced site
cryo-ET: cryo–electron tomography
Env: envelope glycoprotein
FP: fusion peptide
FPPR: fusion peptide–proximal region
HIV-1: human immunodeficiency virus type 1
HR1: heptad repeat 1
HR2: heptad repeat 2
mAb: monoclonal antibody
MFI: mean of fluorescence intensity
MPER: membrane-proximal external region
nAb: neutralizing antibody
nnAb: nonneutralizing antibody
sCD4: soluble CD4
V2: second hypervariable
V3: third hypervariable
wnAb: weakly neutralizing antibody
