# Supplementary figures and images for: CD4 occupancy triggers sequential pre-fusion conformational states of the HIV-1 envelope trimer with relevance for broadly neutralizing antibody activity
Source: PLoS Biol. 2019 Jan 16;17(1):e3000114. doi: 10.1371/journal.pbio.3000114 (PMC6351000; doi:10.1371/journal.pbio.3000114)

S1 Fig

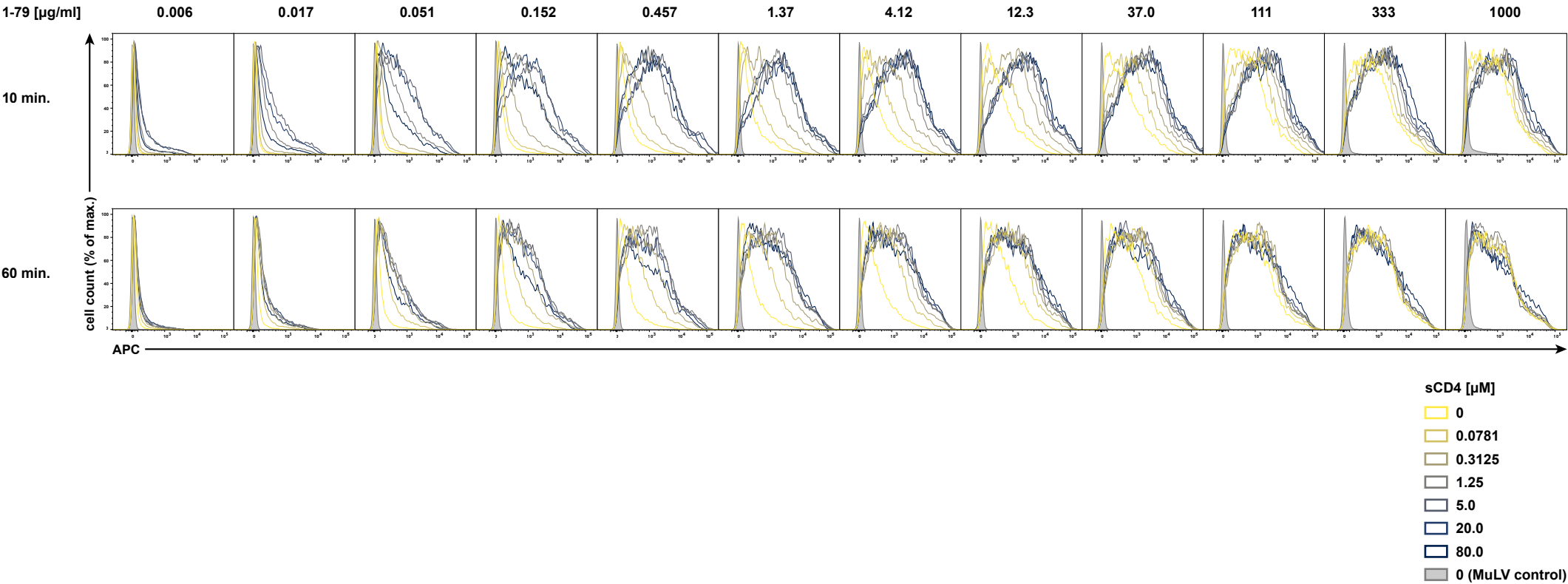

Supplement: S1 Fig — Data related to Fig 1. HEK 293T cells expressing the HIV-1 BaL.01 Env were stained with the indicated concentrations of 1-79 mAb in the presence of increasing sCD4 concentrations for 10 or 60 minutes. Inhibitor binding to MuLV Env–expressing cells in the absence of sCD4 is shown as a measure of nonspecific cell surface staining. Only the fluorescence intensities of live cells are displayed. Env, envelope glycoprotein; HEK, human embryonic kidney; HIV-1, human immunodeficiency virus type 1; mAb, monoclonal antibody; MuLV, murine leukemia virus; sCD4, soluble CD4. (PDF) [file pbio.3000114.s001.pdf]

S2 Fig

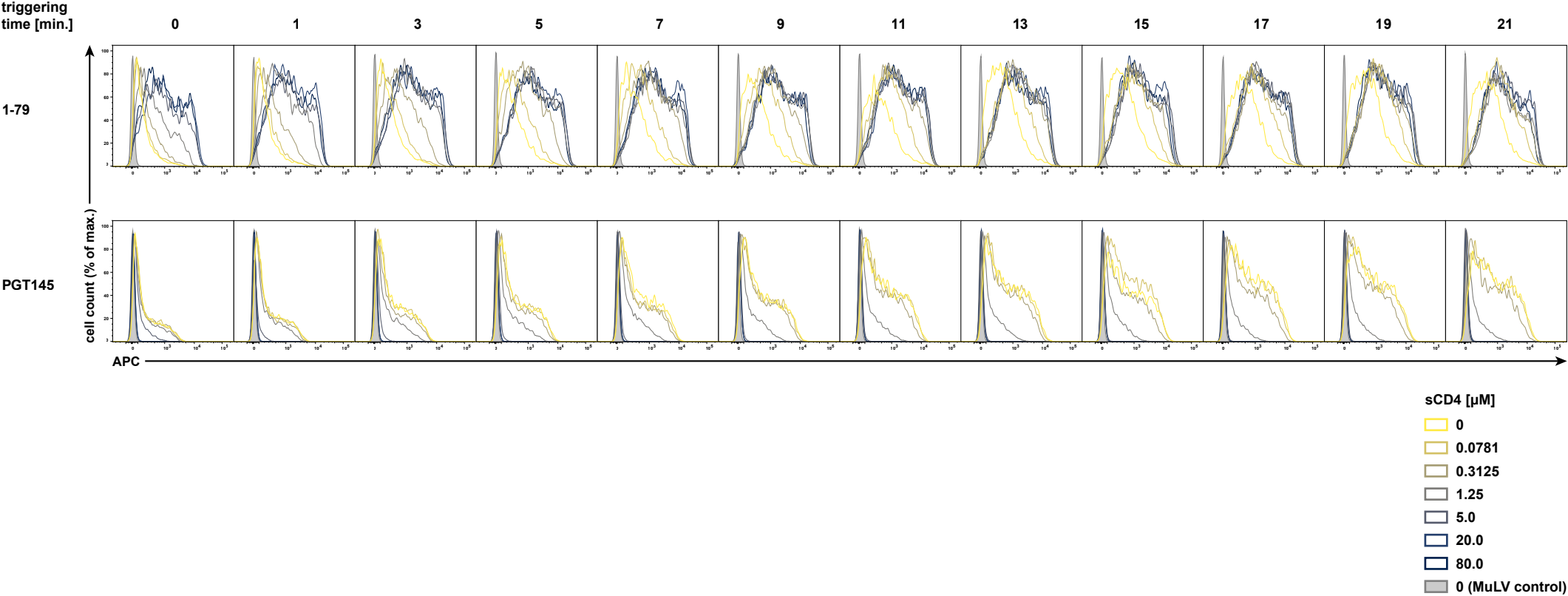

Supplement: S2 Fig — Data related to Fig 2. HEK 293T cells expressing the HIV-1 BaL.01 Env were stained with 0.1 μg/ml of 1-79 or PGT145 mAb in the presence of increasing sCD4 concentrations for up to 21 minutes. Inhibitor binding to MuLV Env–expressing cells in the absence of sCD4 is shown as a measure of nonspecific cell surface staining. Only the fluorescence intensities of live cells are displayed. Env, envelope glycoprotein; HEK, human embryonic kidney; HIV-1, human immunodeficiency virus type 1; mAb, monoclonal antibody; MuLV, murine leukemia virus; sCD4, soluble CD4. (PDF) [file pbio.3000114.s002.pdf]

S3 Fig

CD4-IgG<sub>2</sub>

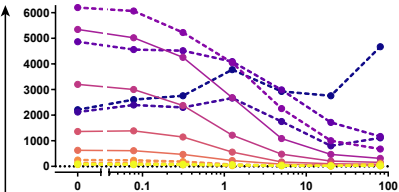

1-79

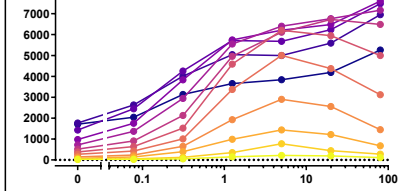

17b

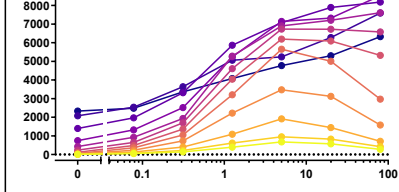

4B3

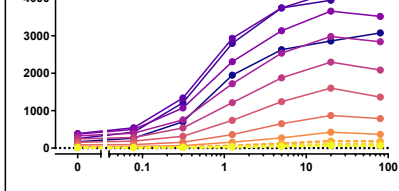

C34-IgG<sub>1</sub>

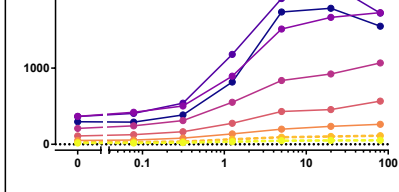

PGT145

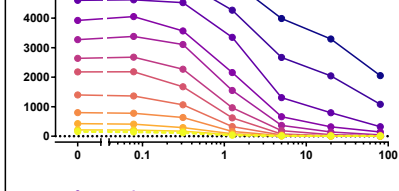

2G12

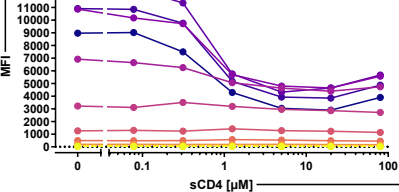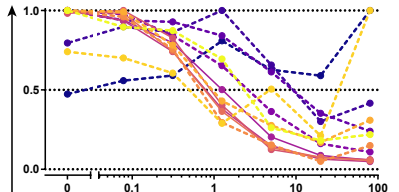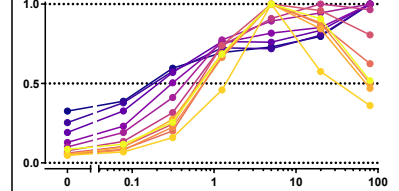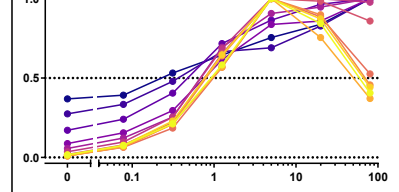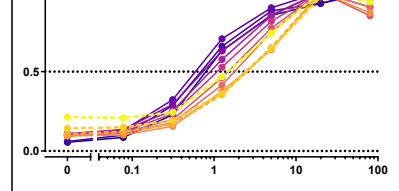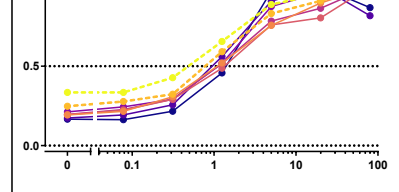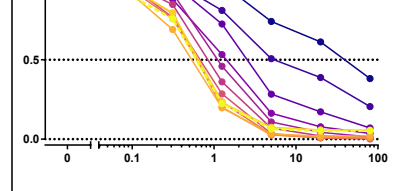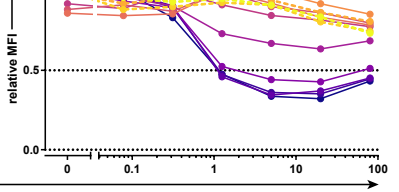

PGT128

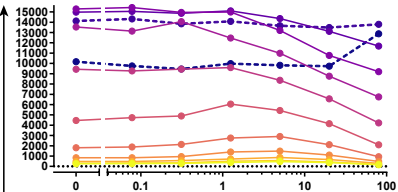

PGT121

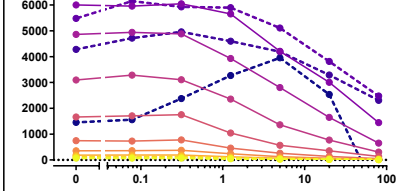

PGT135

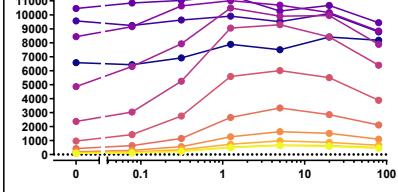

PGT151

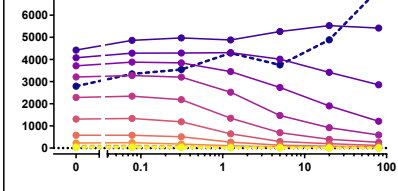

VRC34.01

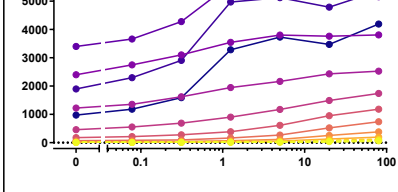

4E10

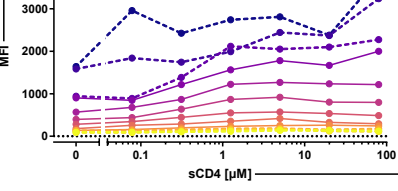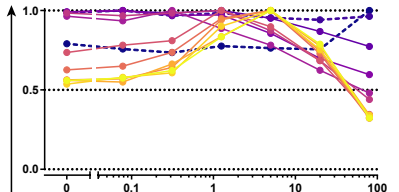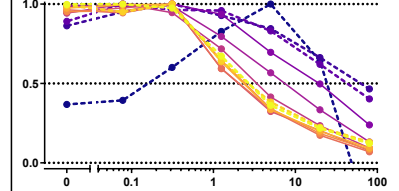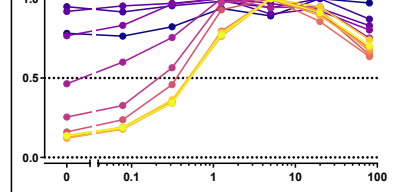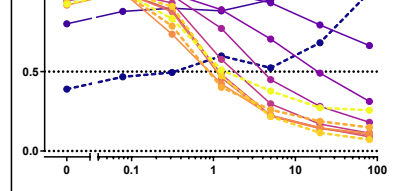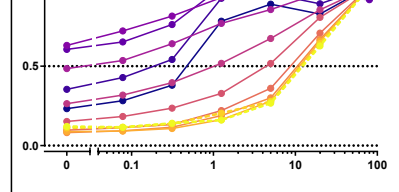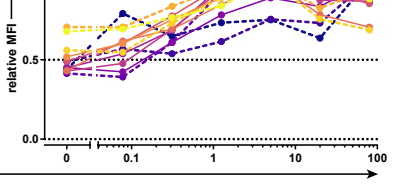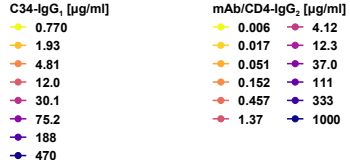

Supplement: S3 Fig — Data related to Fig 3 and S4 Fig. Env-directed inhibitors were titrated in the on-cell sCD4 triggering assay with a 20-minute triggering step. MFI staining curves and relative MFI staining curves were derived as described in Fig 1. MFI staining curves with less than 10-fold higher signal over MuLV background at the peak and the corresponding normalized MFI staining curves are plotted as dashed curves. Data represent a single experiment. Env, envelope glycoprotein; MFI, mean of fluorescence intensity; MuLV, murine leukemia virus; sCD4, soluble CD4. (PDF) [file pbio.3000114.s003.pdf]

S4 Fig

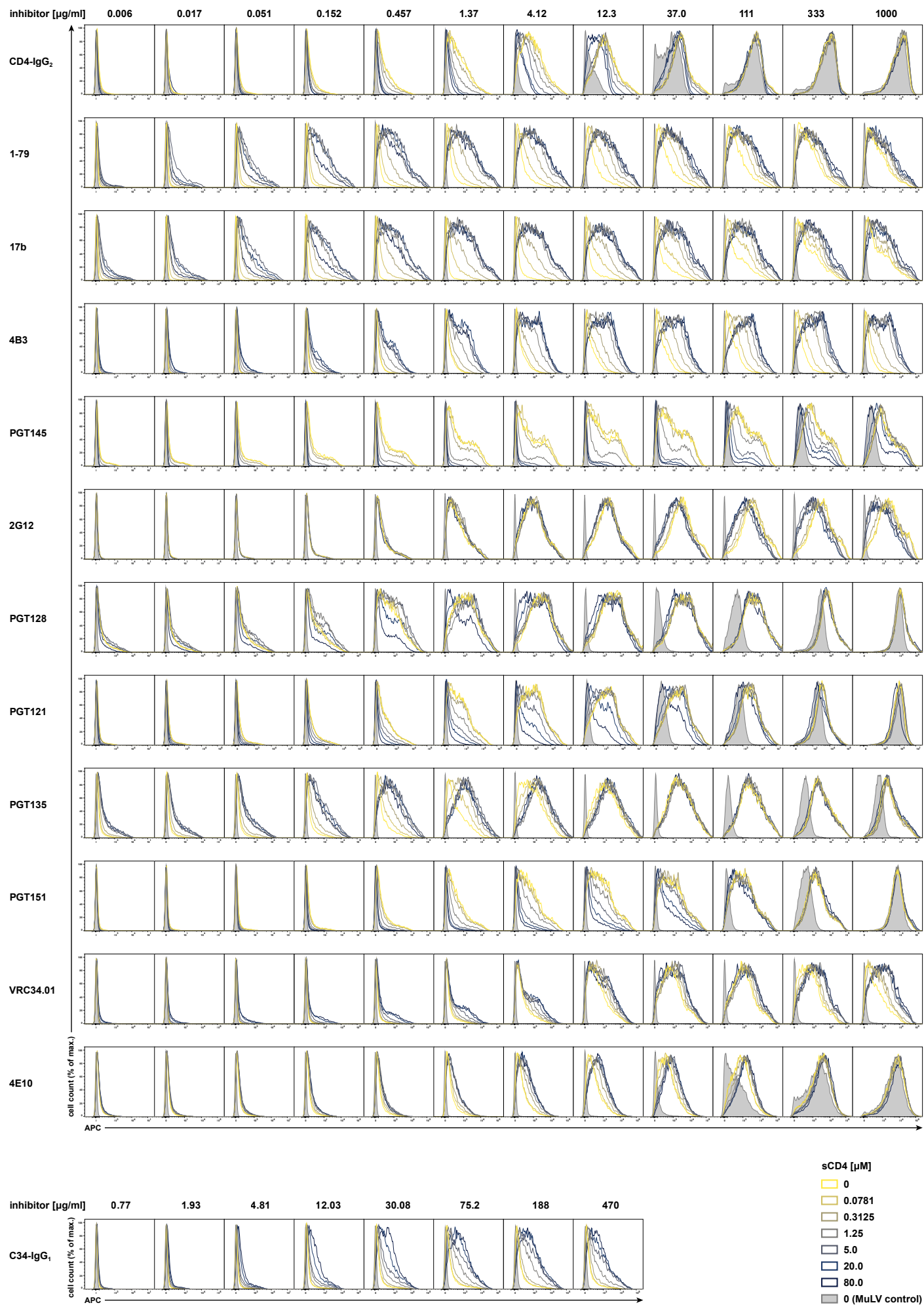

Supplement: S4 Fig — Data related to Fig 3 and S3 Fig. HEK 293T cells expressing the HIV-1 BaL.01 Env were stained with various Env-directed inhibitors at the indicated concentrations in the presence of increasing concentrations of sCD4 for 20 minutes. Inhibitor binding to MuLV Env–expressing cells in the absence of sCD4 is shown as a measure of nonspecific cell surface staining. Only the fluorescence intensities of live cells are displayed. Env, envelope glycoprotein; HEK, human embryonic kidney; HIV-1, human immunodeficiency virus type 1; MuLV, murine leukemia virus; sCD4, soluble CD4. (PDF) [file pbio.3000114.s004.pdf]

S5 Fig

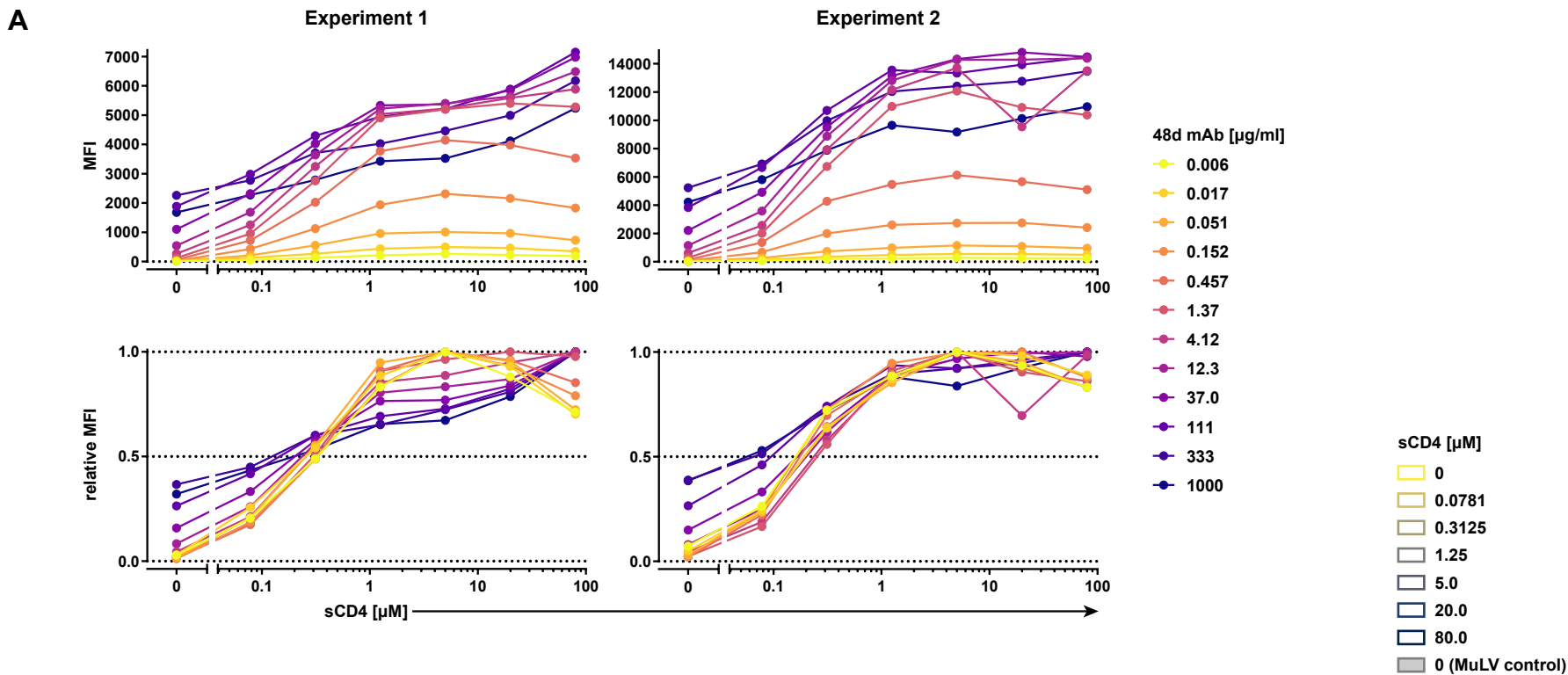

**B**

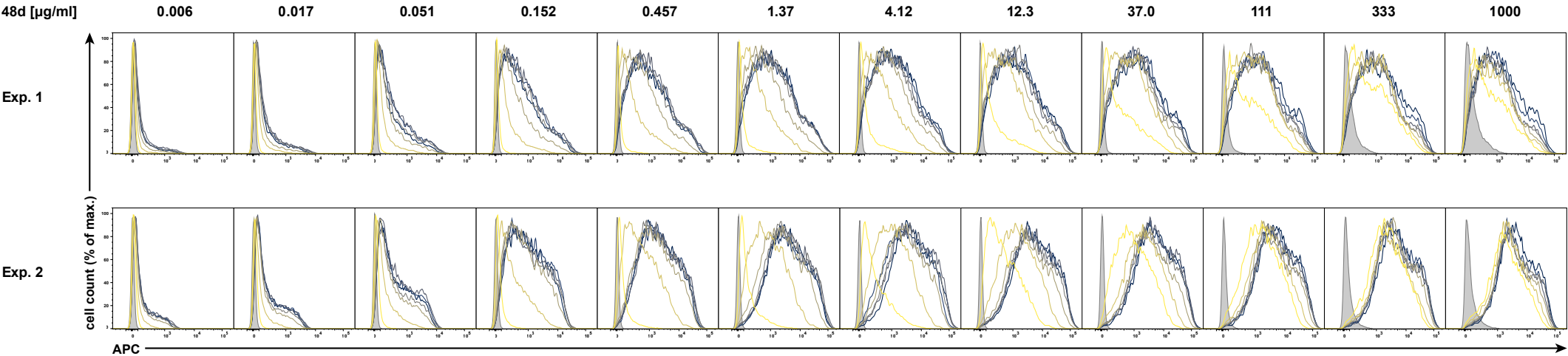

Supplement: S5 Fig — HEK 293T cells expressing the HIV-1 BaL.01 Env were stained with the indicated concentrations of the CD4i mAb 48d in the presence of increasing sCD4 concentrations for 20 minutes. (A) MFI staining curves and relative MFI staining curves were derived as described in Fig 1. (B) Histogram plots corresponding to data shown in panel A. Inhibitor binding to MuLV Env–expressing cells in the absence of sCD4 is shown as a measure of nonspecific cell surface staining. Only the fluorescence intensities of live cells are displayed. The experiments were performed on two different days using different cell batches. CD4i, CD4-induced site; Env, envelope glycoprotein; HEK, human embryonic kidney; HIV-1, human immunodeficiency virus type 1; mAb, monoclonal antibody; MFI, mean of fluorescence intensity; MuLV, murine leukemia virus; sCD4, soluble CD4. (PDF) [file pbio.3000114.s005.pdf]

S6 Fig

A

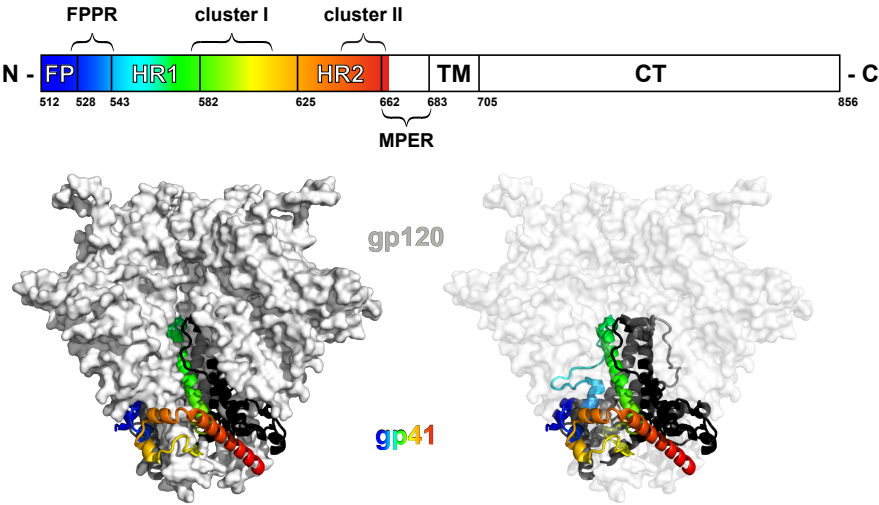

B

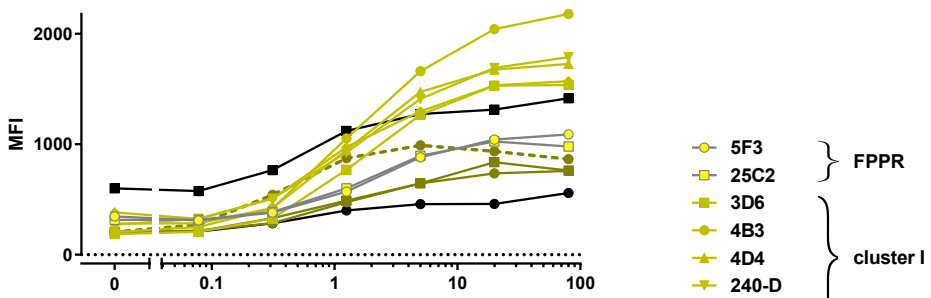

C

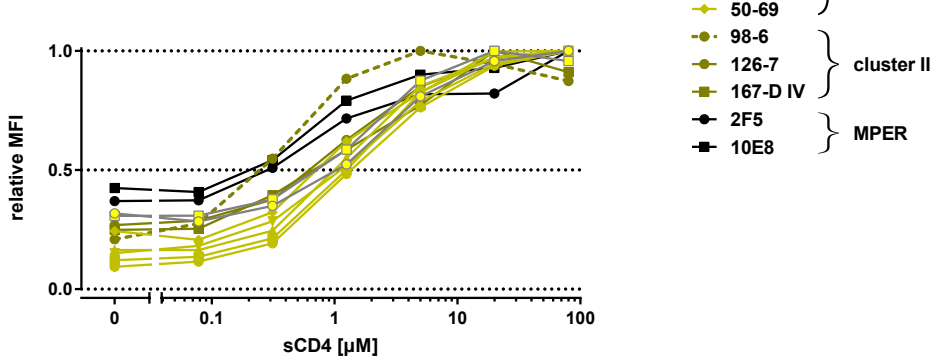

D

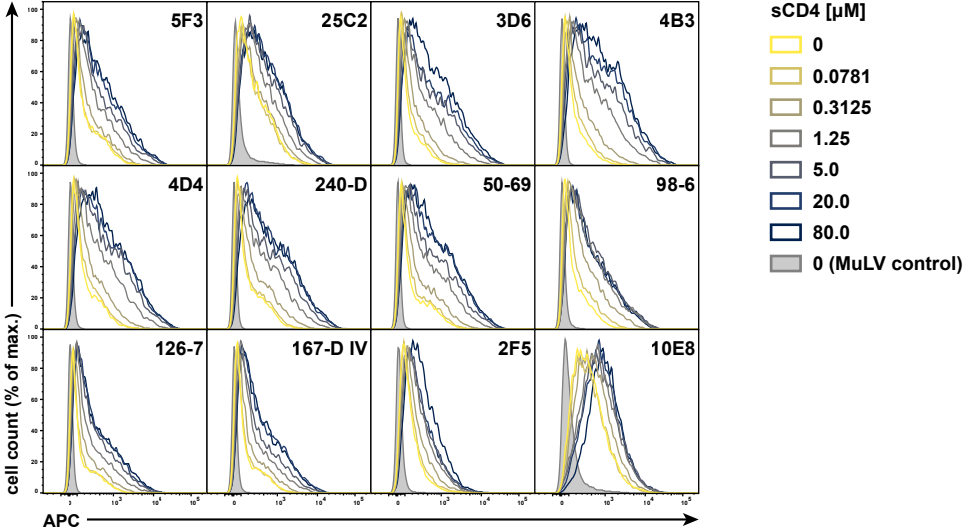

Supplement: S6 Fig — (A) Domain organization of HIV-1 gp41 with locations of major antibody epitope clusters (HXB2 numbering). Depicted is a crystal structure of stabilized soluble X1193.c1 Env trimer (PDB ID 5FYJ). All three gp120 subunits are in gray; three gp41 subunits are in either black, dark gray, or rainbow (blue to red). On-cell sCD4 triggering of BaL.01 Env was carried out with inhibitors at a fixed concentration of 10 μg/ml with a 20-minute triggering step. MFI (B) and relative MFI (C) staining curves of gp41-directed mAbs in BaL.01 on-cell sCD4 triggering assay were obtained as described in Fig 1. (D) Histogram plots corresponding to data shown in panels B and C. Inhibitor binding to MuLV Env–expressing cells in the absence of sCD4 is shown as a measure of nonspecific cell surface staining. Only the fluorescence intensities of live cells are displayed. Data represent a single experiment. CT, cytoplasmic tail; Env, envelope glycoprotein; HIV-1, human immunodeficiency virus type 1; mAb, monoclonal antibody; MFI, mean of fluorescence intensity; MuLV, murine leukemia virus; PDB, Protein Data Bank; sCD4, soluble CD4; TM, transmembrane region. (PDF) [file pbio.3000114.s006.pdf]

S7 Fig

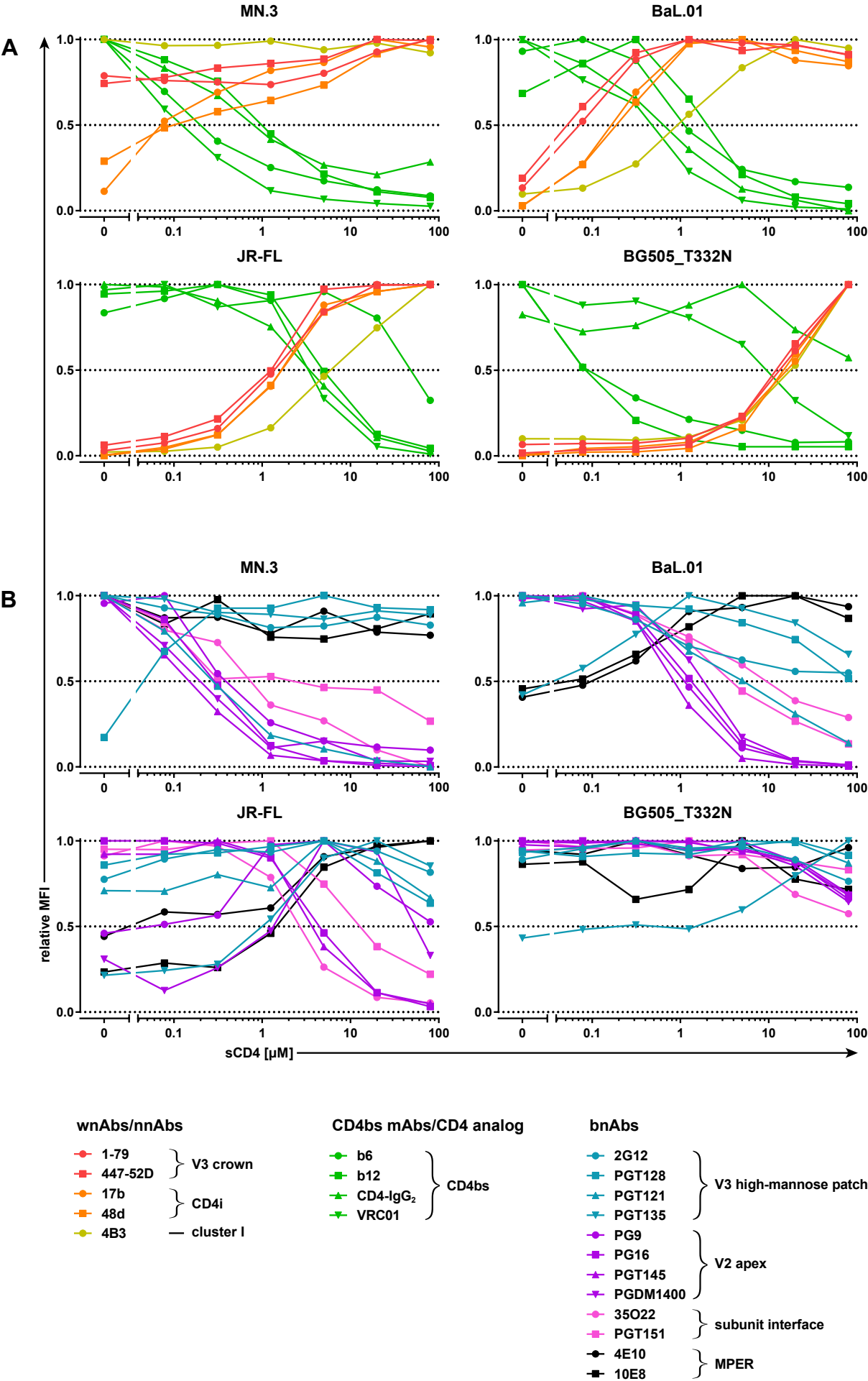

Supplement: S7 Fig — Related to Fig 4 and S8 Fig. MFI staining curves of selected gp120- and gp41-directed inhibitors from the on-cell sCD4 triggering assay with HIV-1 MN.3, BaL.01, JR-FL, and BG505_T332N Envs depicted in Fig 4 were normalized to their maxima. For clarity, the resulting relative MFI staining curves are shown separately for wnAbs/nnAbs and CD4bs-directed reagents (A) and for bnAbs (B). BG505_T332N, BG505.W6M.ENV.C2_T332N; bnAb, broadly neutralizing antibody; CD4bs, CD4 binding site; Env, envelope glycoprotein; HIV-1, human immunodeficiency virus type 1; MFI, mean of fluorescence intensity; nnAb, nonneutralizing antibody; sCD4, soluble CD4; wnAb, weakly neutralizing antibody. (PDF) [file pbio.3000114.s007.pdf]

S8 Fig

MN.3

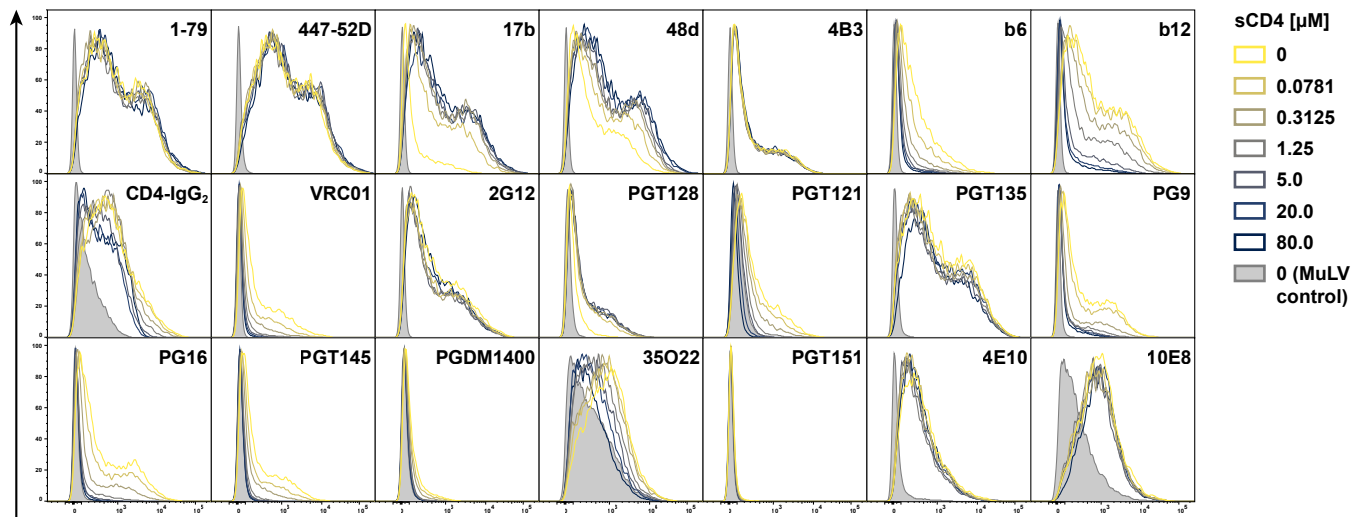

BaL.01

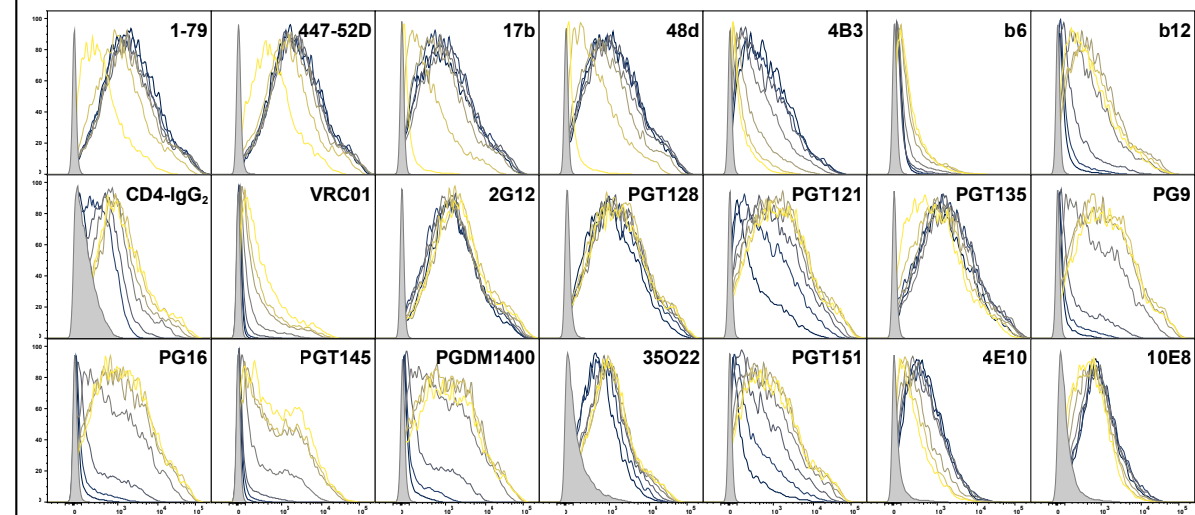

JR-FL

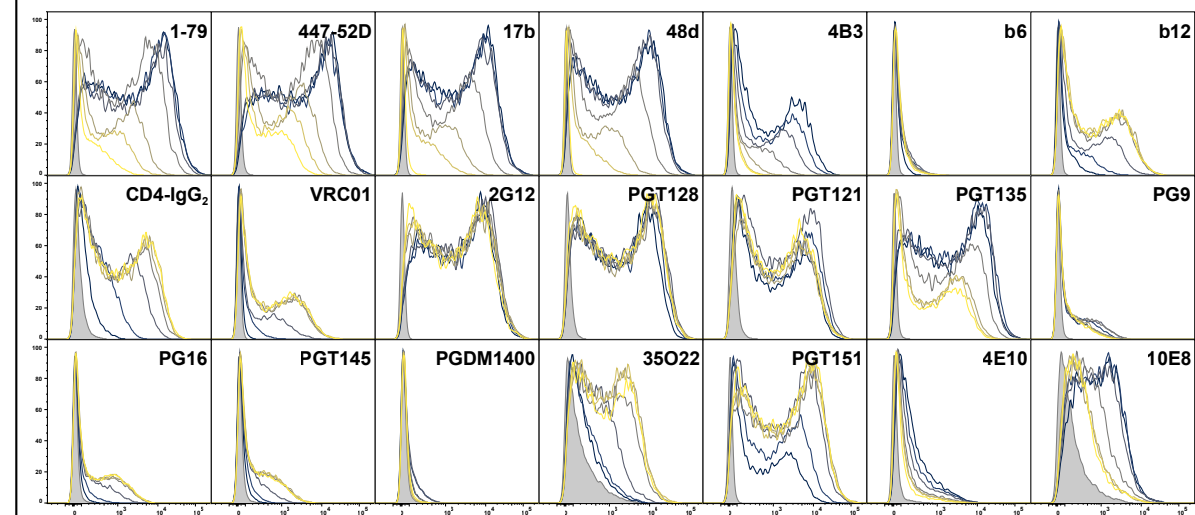

BG505\_ T332N

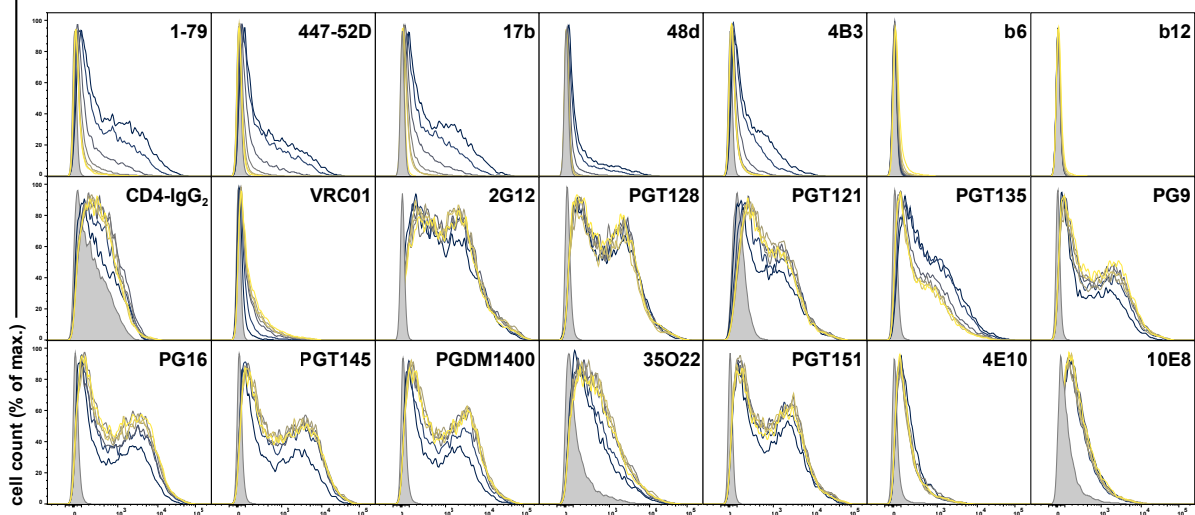

Supplement: S8 Fig — Related to Fig 4 and S7 Fig. Binding of a panel of HIV-1 Env–directed inhibitors at 10 μg/ml in the presence of increasing sCD4 concentrations to HEK 293T cells expressing the HIV-1 MN.3, BaL.01, JR-FL, and BG505_T332N Envs. Inhibitor binding to MuLV Env–expressing cells in the absence of sCD4 is shown as a measure of nonspecific cell surface staining. Only the fluorescence intensities of live cells are displayed. BG505_T332N, BG505.W6M.ENV.C2_T332N; Env, envelope glycoprotein; HEK, human embryonic kidney; HIV-1, human immunodeficiency virus type 1; MuLV, murine leukemia virus; sCD4, soluble CD4. (PDF) [file pbio.3000114.s008.pdf]

S9 Fig

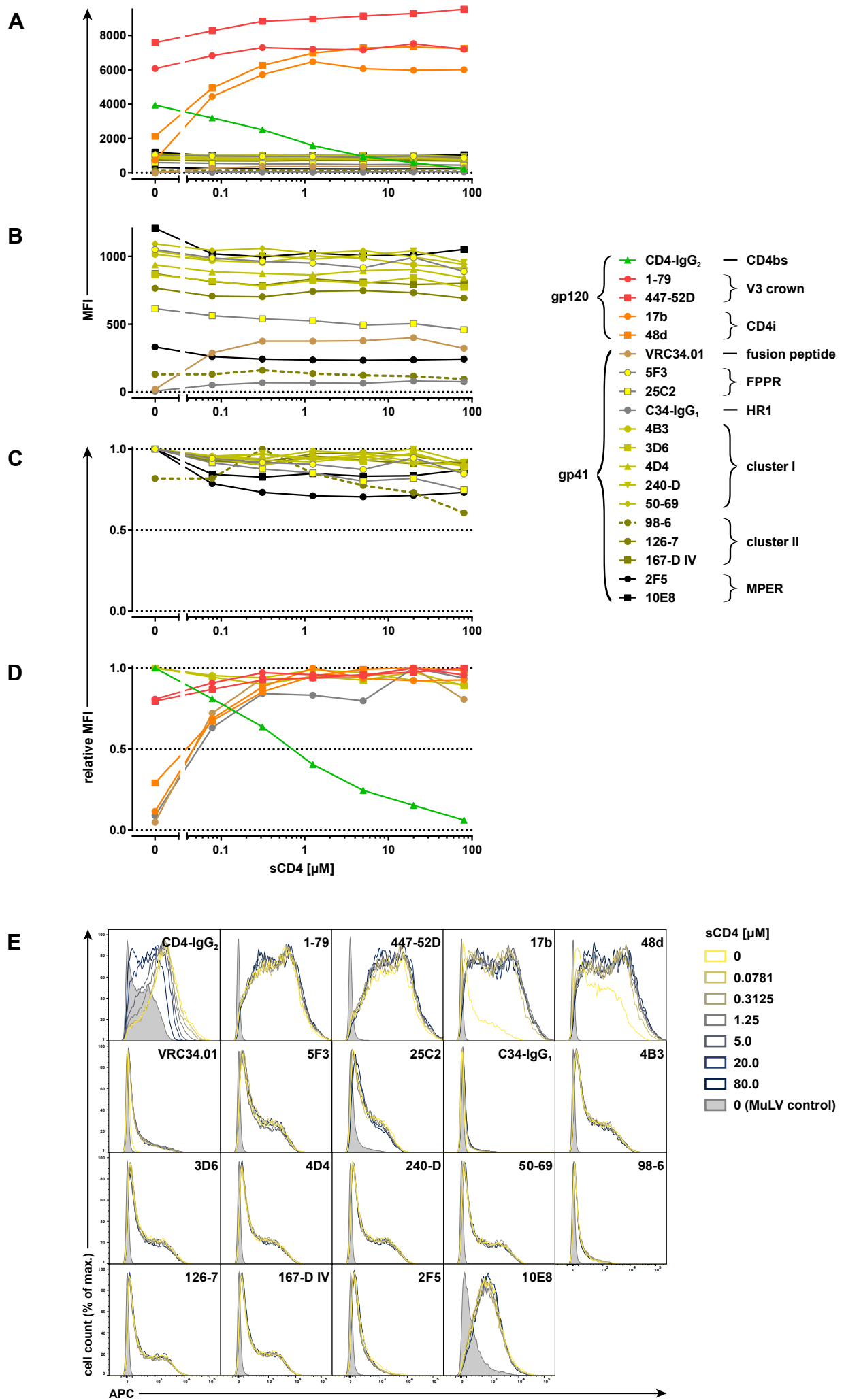

Supplement: S9 Fig — (A, B) MFI staining curves of selected gp120- and gp41-directed inhibitors from on-cell sCD4 triggering of MN.3 Env were obtained as in Fig 4. MFI staining curves are shown for all (A) or only gp41-directed inhibitors (B) for clarity. (C, D) MFI staining curves normalized to their maxima (relative MFI staining curves) are shown for gp41-targeting mAbs (C) and selected gp120- and gp41-directed inhibitors (D). (E) Underlying flow cytometry data in the form of histogram plots. Inhibitor binding to MuLV Env–expressing cells in the absence of sCD4 is shown as a measure of nonspecific cell surface staining. Only the fluorescence intensities of live cells are displayed. Data represent a single experiment. Env, envelope glycoprotein; mAb, monoclonal antibody; MFI, mean of fluorescence intensity; MuLV, murine leukemia virus; sCD4, soluble CD4. (PDF) [file pbio.3000114.s009.pdf]

S10 Fig

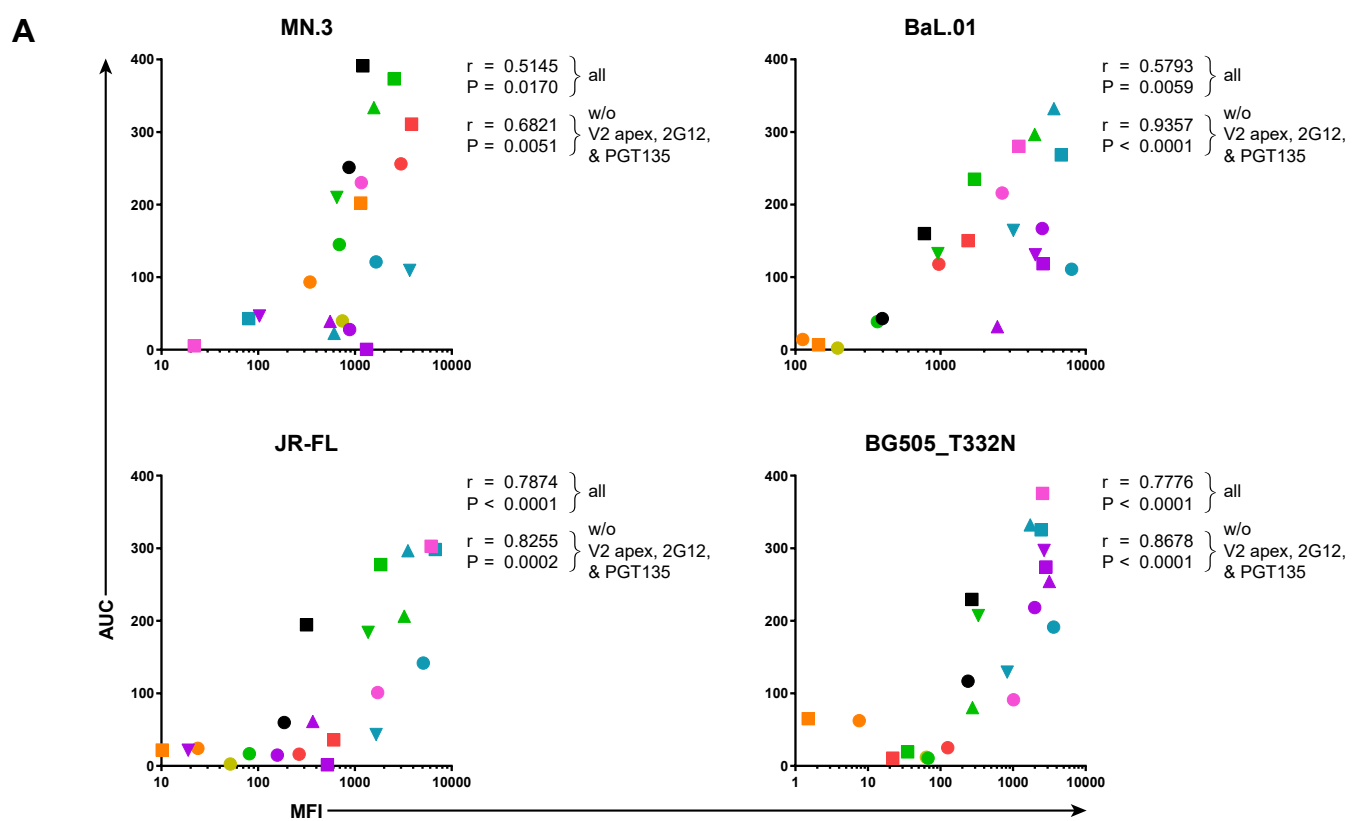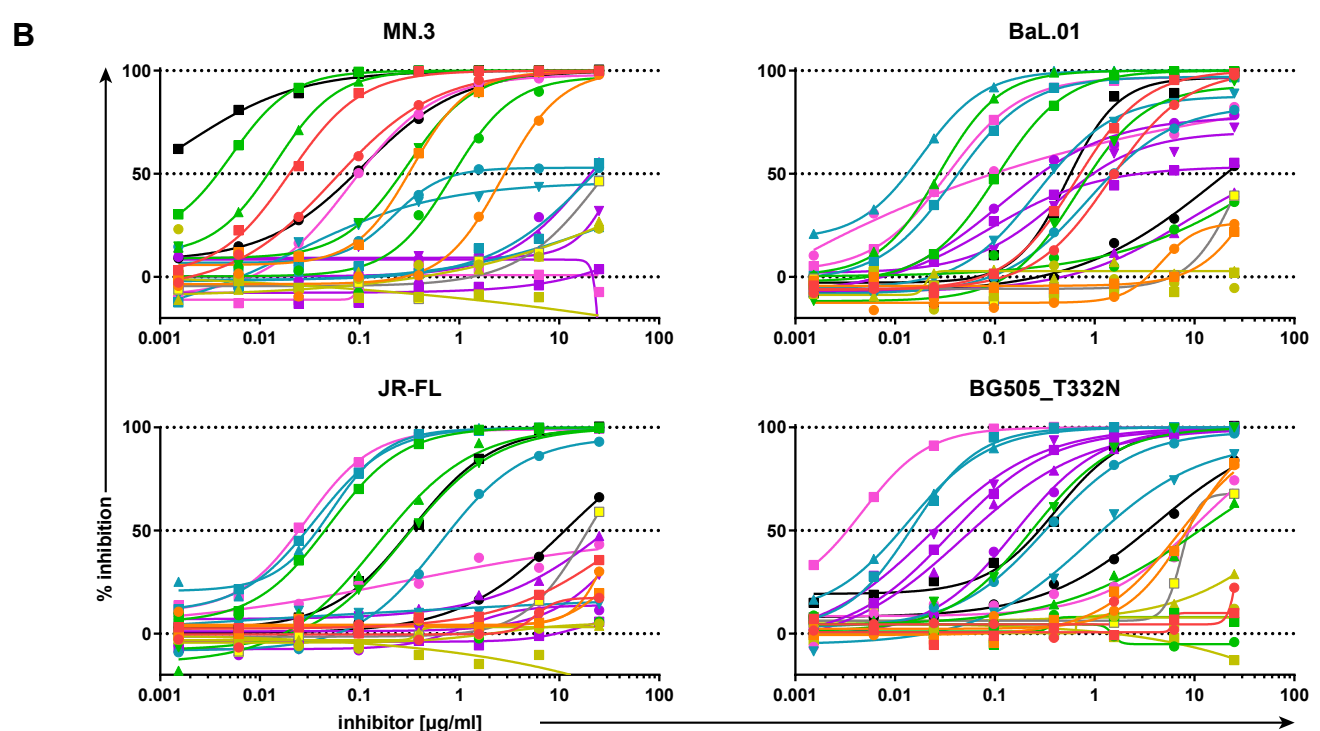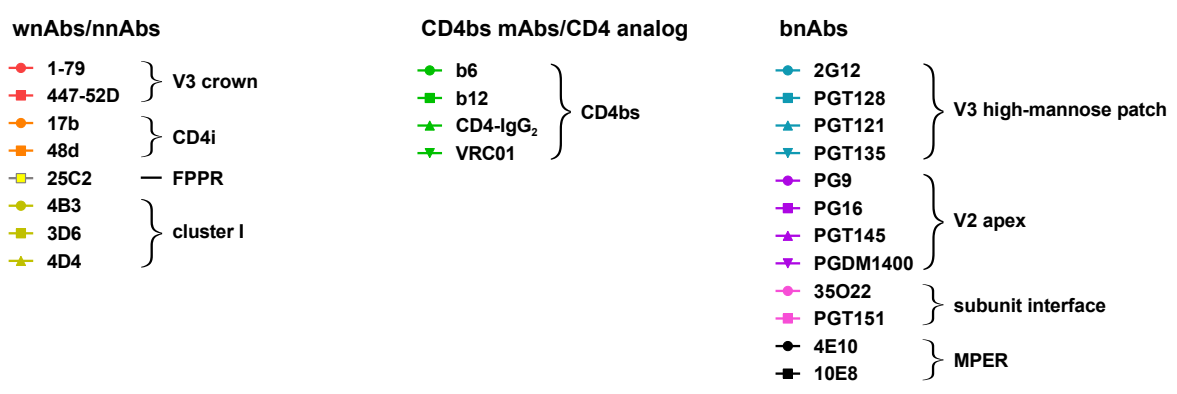

Supplement: S10 Fig — Related to Fig 5. (A) Correlation between the MFI of inhibitor binding to the native, CD4-unbound MN.3, BaL.01, JR-FL, and BG505_T332N Env (MFI data from Fig 4) and the AUC derived from neutralization data of the corresponding pseudovirus shown in panel B. Pearson’s r coefficients and respective P values were calculated taking into consideration either all probed inhibitors or all inhibitors excluding V2 apex bnAbs (PG9, PG16, PGT145, PGDM1400), 2G12, and PGT135. (B) Neutralization assay of HIV-1 pseudovirus carrying MN.3, BaL.01, JR-FL, and BG505_T332N Envs with multiple cases of incomplete neutralization. Data represent a single experiment. AUC, area under the inhibition curve; BG505_T332N, BG505.W6M.ENV.C2_T332N; bnAb, broadly neutralizing antibody; Env, HIV-1 envelope glycoprotein; HIV-1, human immunodeficiency virus type 1; MFI, mean of fluorescence intensity; MuLV, murine leukemia virus; sCD4, soluble CD4; V2, second hypervariable. (PDF) [file pbio.3000114.s010.pdf]

S11 Fig

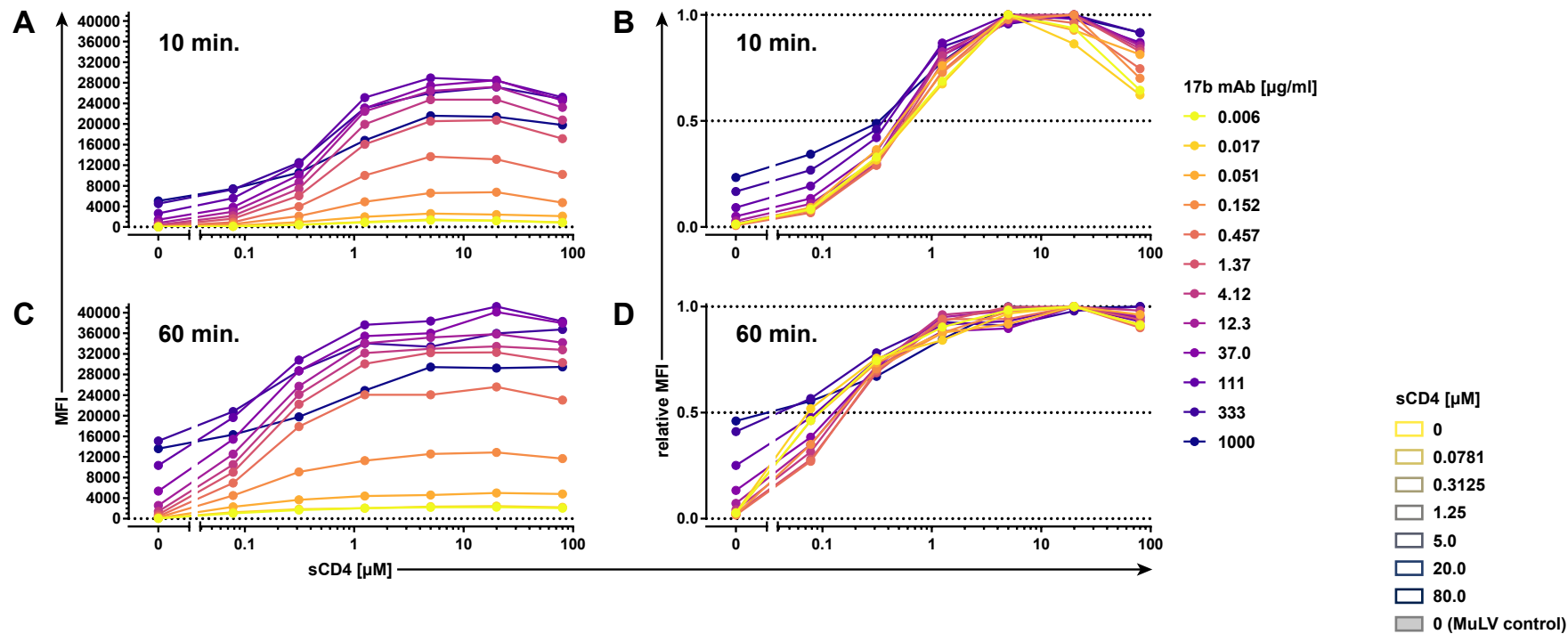

E

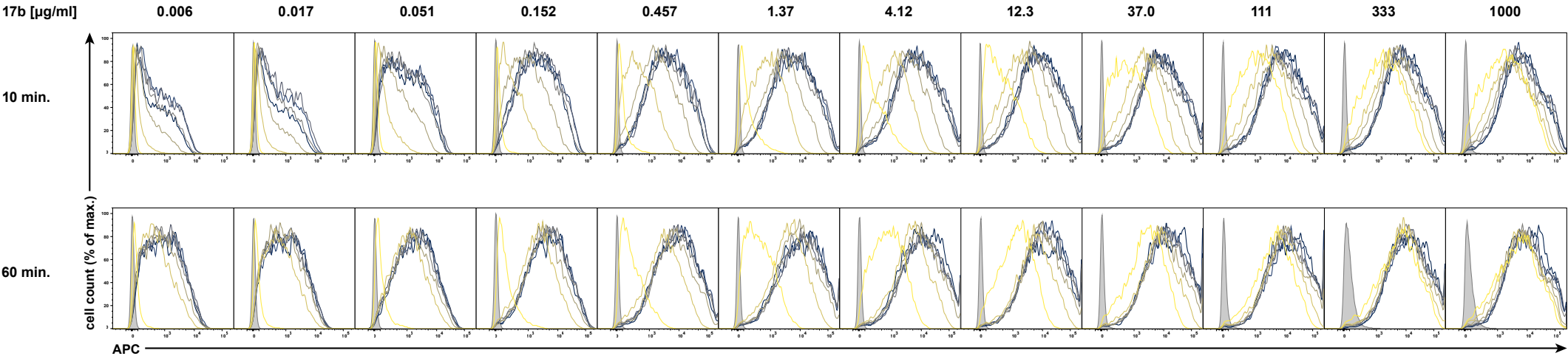

Supplement: S11 Fig — HEK 293T cells expressing the HIV-1 BaL.01 SOS Env were stained with the indicated concentrations of the CD4i mAb 17b in the presence of increasing sCD4 concentrations for 10 minutes (A, B) or 60 minutes (C, D). MFI staining curves (A, C) and relative MFI staining curves (B, D) were derived as described in Fig 1. (E) Histogram plots corresponding to data shown in panels A–D. Inhibitor binding to MuLV Env–expressing cells in the absence of sCD4 is shown as a measure of nonspecific cell surface staining. Only the fluorescence intensities of live cells are displayed. Data represent a single experiment. CD4i, CD4-induced site; Env, envelope glycoprotein; HEK, human embryonic kidney; HIV-1, human immunodeficiency virus type 1; mAb, monoclonal antibody; MFI, mean of fluorescence intensity; MuLV, murine leukemia virus; sCD4, soluble CD4. (PDF) [file pbio.3000114.s011.pdf]

S12 Fig

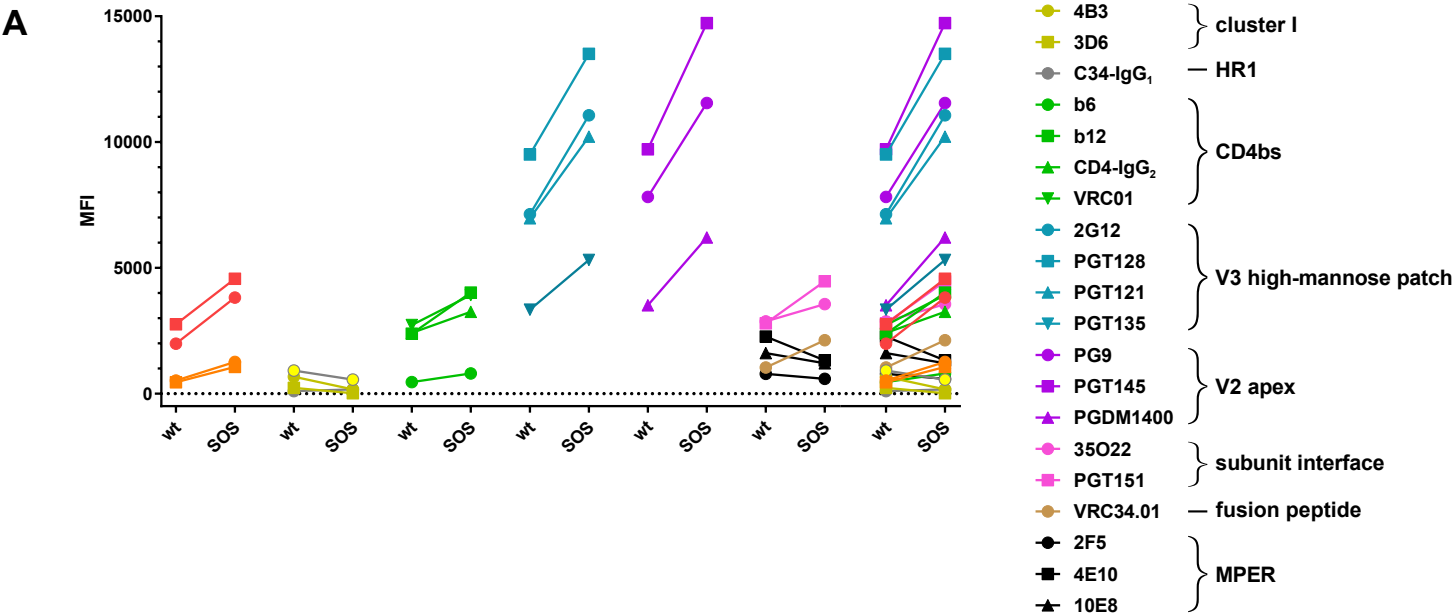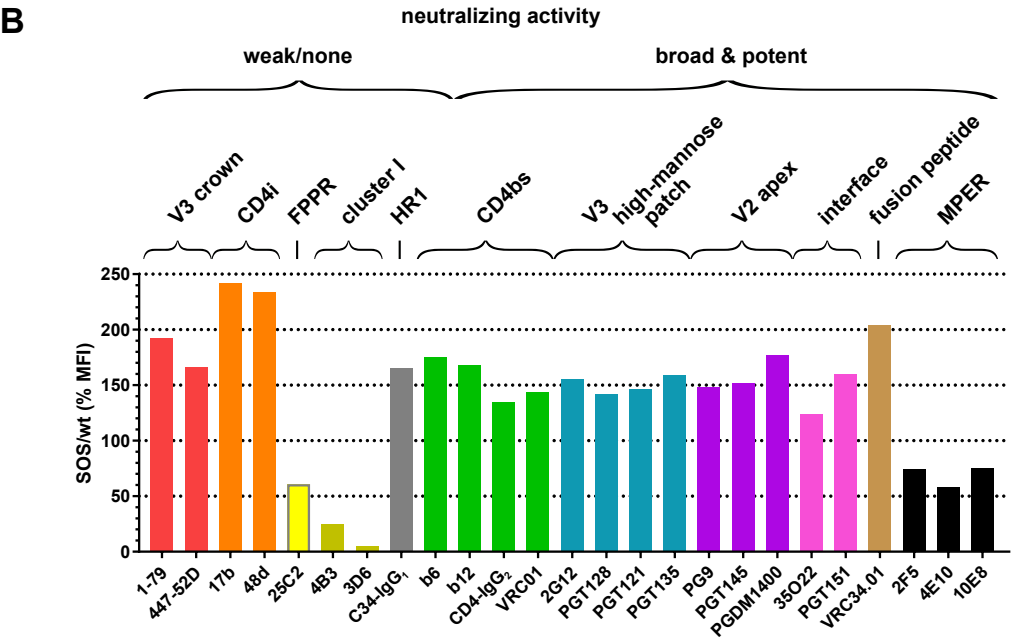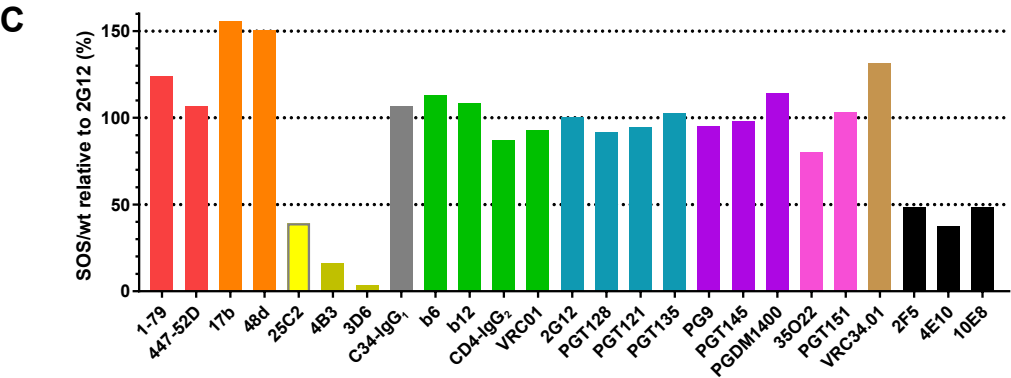

Supplement: S12 Fig — HEK 293T cells expressing the MuLV, HIV-1 BaL.01, or HIV-1 BaL.01 SOS Env were stained with 5 μg/ml of the indicated HIV-1 Env–directed inhibitors. The amount of antibody bound to the cell surface was quantified by flow cytometry. Dead cells were excluded from analysis, and background signal from the MuLV Env–expressing control cells was subtracted from the signal of the HIV-1 Env–expressing cells. The resulting MFI values are depicted in panel A and the derived BaL.01 SOS/wild-type MFI ratios in panel B. To correct for general difference in expression between the two Envs and to allow for an easier visual readout of binding differences, the MFI ratios from panel B were normalized to the MFI ratio of mAb 2G12 (C). Env, envelope glycoprotein; HEK, human embryonic kidney; HIV-1, human immunodeficiency virus type 1; mAb, monoclonal antibody; MFI, mean of fluorescence intensity; MuLV, murine leukemia virus. (PDF) [file pbio.3000114.s012.pdf]

S13 Fig

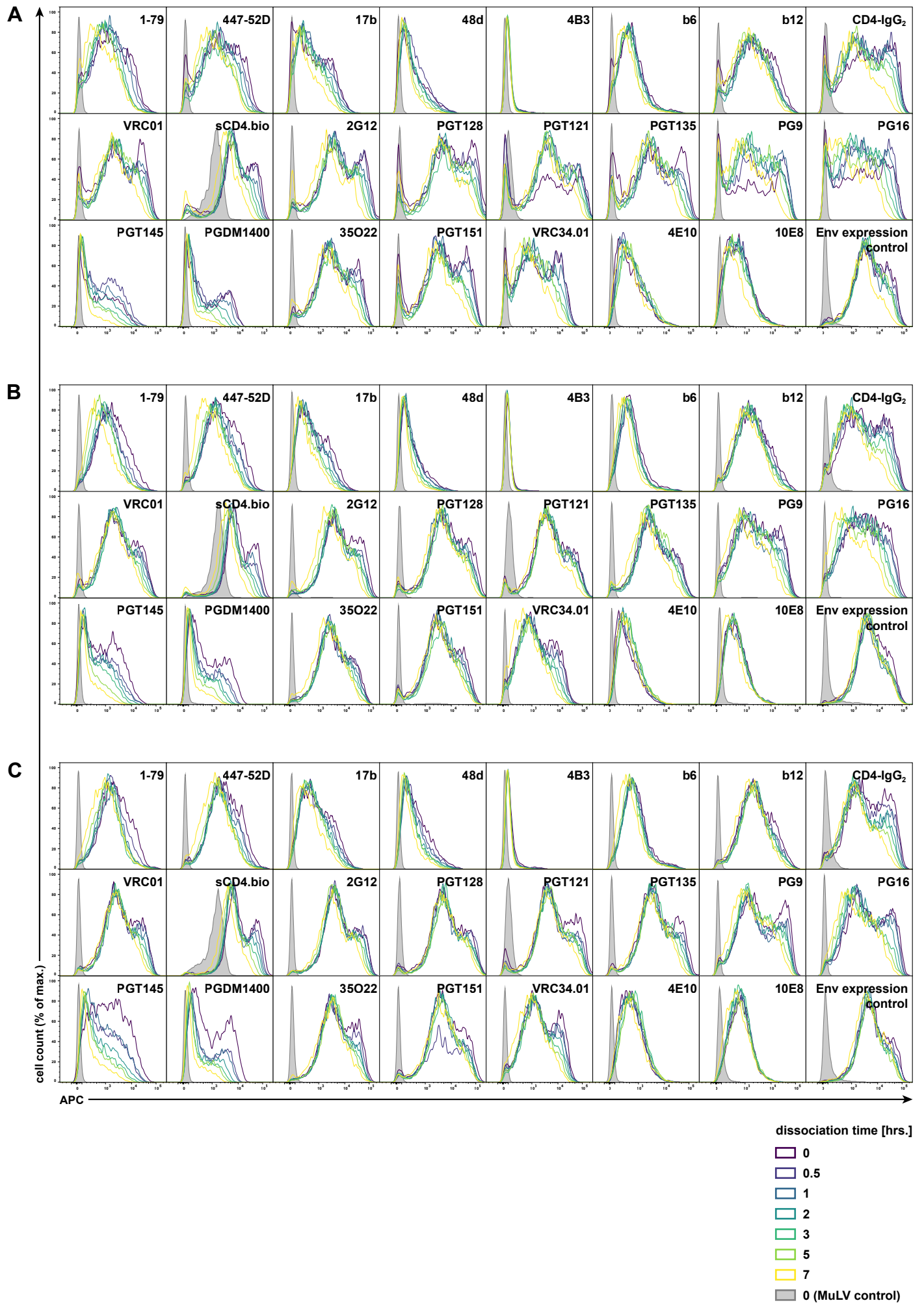

Supplement: S13 Fig — Related to Fig 6. BaL.01 SOS Env–expressing HEK 293T cells were stained with 5 μM biotinylated sCD4 or 10 μg/ml of other HIV-1 inhibitors that were then allowed to dissociate at 37 °C until the cells were chemically fixed at the indicated time points. Cells that were stained with 10 μg/ml of 2G12 mAb only after fixing served as an “Env expression control” to depict the changing amount of Env on the cell surface during the experiment. (A, B, C) Histogram plots from three independent experiments. Inhibitor binding to MuLV Env–expressing cells at t = 0 is shown as a measure of nonspecific cell surface staining. Only the fluorescence intensities of live cells are displayed. Env, envelope glycoprotein; HEK, human embryonic kidney; HIV-1, human immunodeficiency virus type 1; mAb, monoclonal antibody; MFI, mean of fluorescence intensity; MuLV, murine leukemia virus; sCD4, soluble CD4. (PDF) [file pbio.3000114.s013.pdf]

S14 Fig

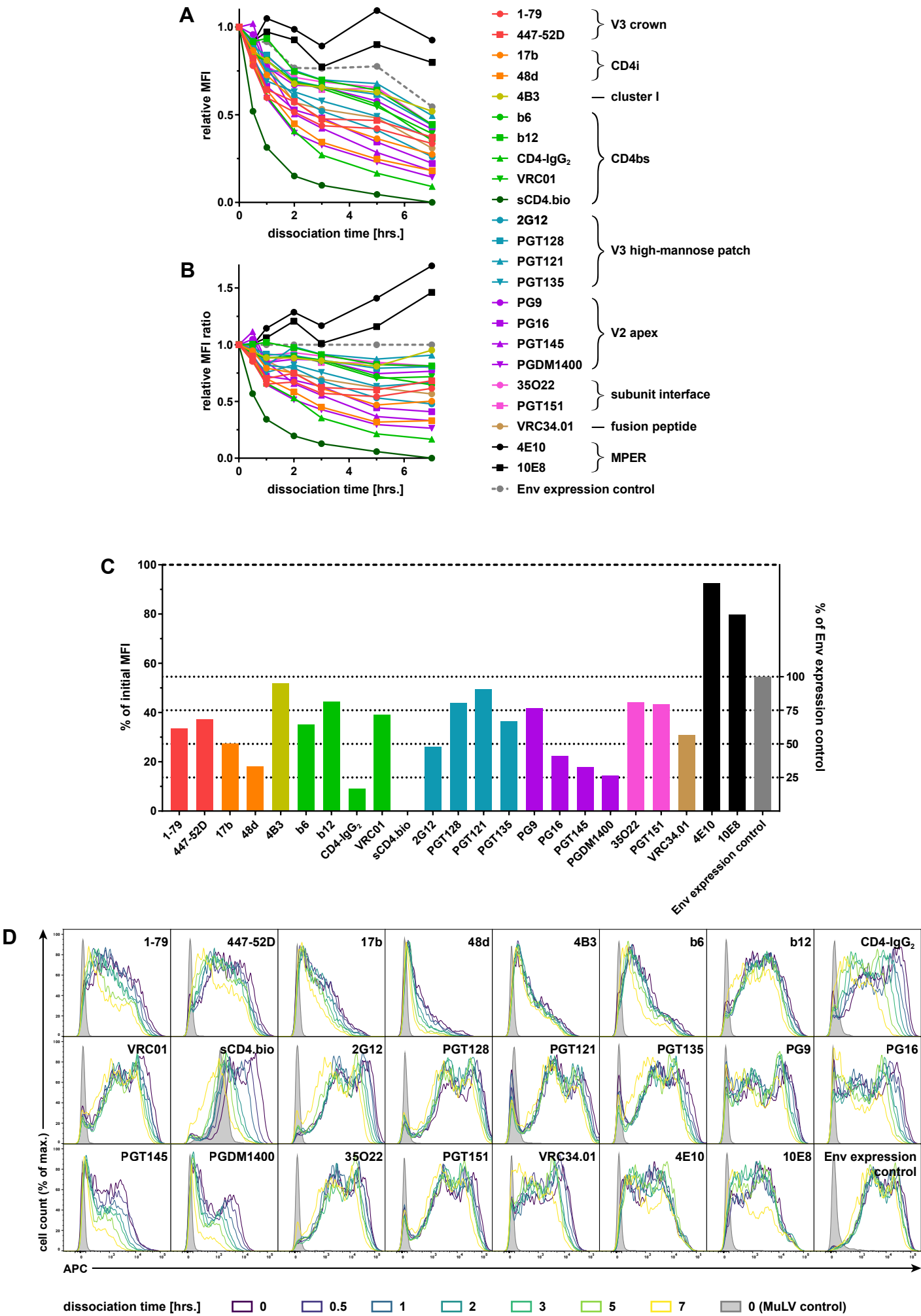

Supplement: S14 Fig — Data from the dissociation assay were acquired and processed as described in Fig 6. (A) MFI values for each inhibitor and Env expression control were normalized to the dissociation time t = 0. (B) The resulting relative MFI values for each inhibitor were divided by the relative MFI value of the Env expression control for the respective time point. (C) Relative MFI values for each inhibitor and Env expression control at dissociation time t = 7 hours. (D) Underlying flow cytometry data in the form of histogram plots. Inhibitor binding to MuLV Env–expressing cells at t = 0 is shown as a measure of nonspecific cell surface staining. Only the fluorescence intensities of live cells are displayed. Data represent a single experiment. Env, envelope glycoprotein; MFI, mean of fluorescence intensity; MuLV, murine leukemia virus. (PDF) [file pbio.3000114.s014.pdf]

S15 Fig

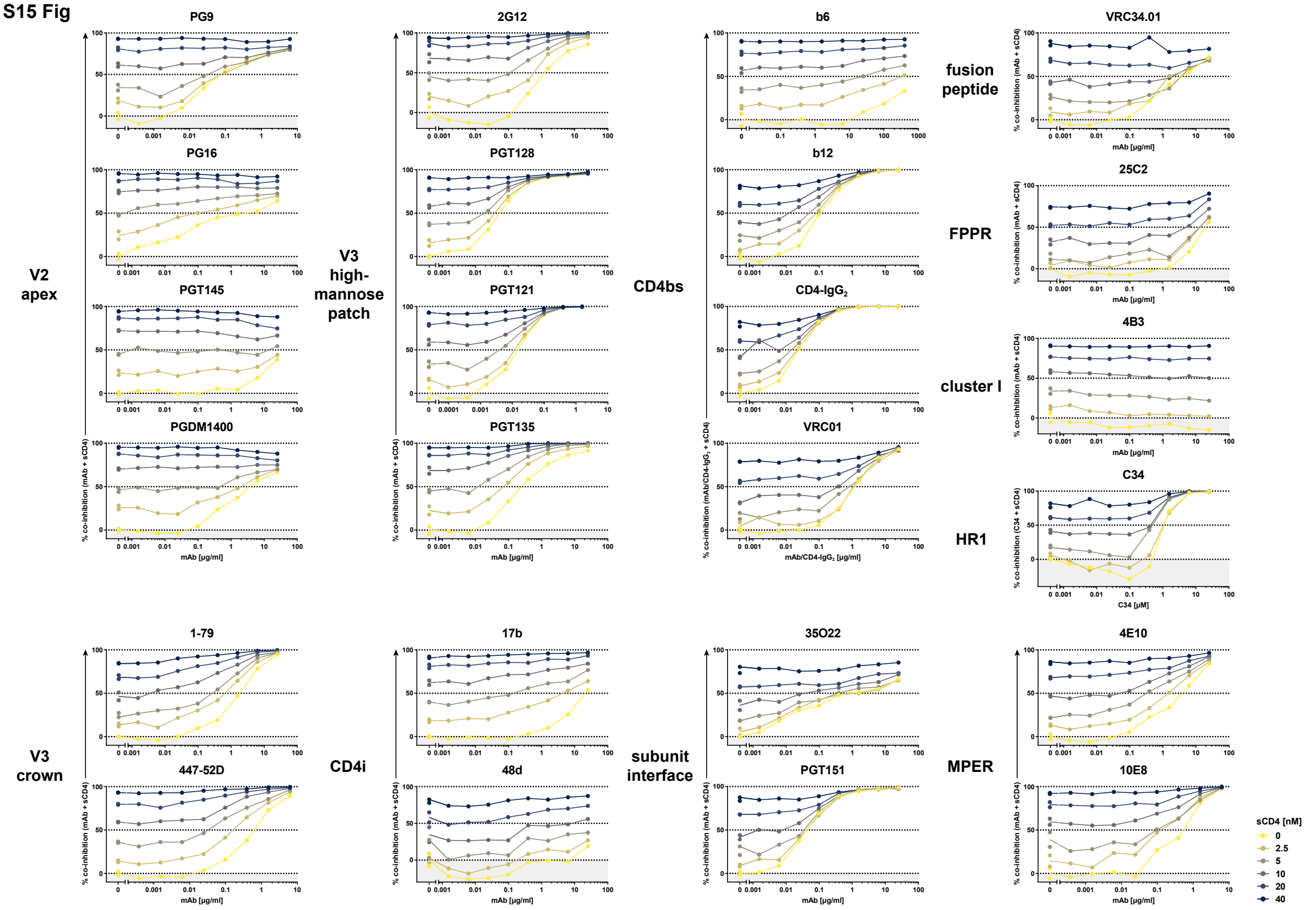

Supplement: S15 Fig — Related to Fig 7 and S16 Fig. Neutralization potency of different Env-directed inhibitors against BaL.01 pseudovirus was assayed in the presence of increasing sCD4 concentrations. The percent coinhibition value was set relative to the infectivity measured with no inhibitor present. Data depict a single experiment or a representative experiment of two to three conducted. Env, envelope glycoprotein; sCD4, soluble CD4. (PDF) [file pbio.3000114.s015.pdf]

S16 Fig

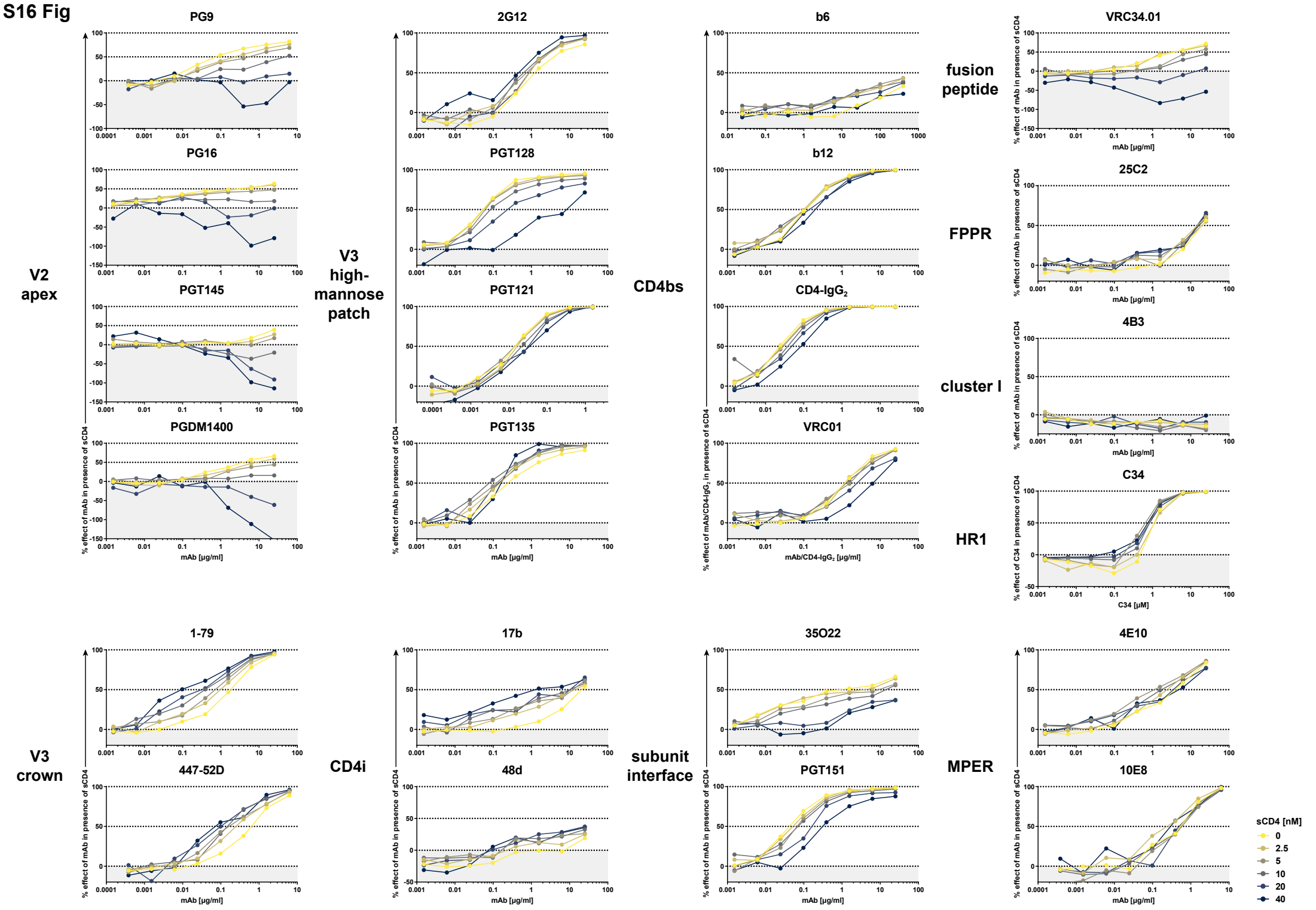

Supplement: S16 Fig — Related to Fig 7 and S15 Fig. Neutralization potency of different Env-directed inhibitors against BaL.01 pseudovirus was assayed in the presence of increasing sCD4 concentrations. The percent effect value of an inhibitor was set relative to the infectivity measured with sCD4 at the specified concentration in the inhibitor absence. Data depict a single experiment or a representative experiment of two to three conducted. Env, envelope glycoprotein; sCD4, soluble CD4. (PDF) [file pbio.3000114.s016.pdf]
