# Supplementary material for: CD4 occupancy triggers sequential pre-fusion conformational states of the HIV-1 envelope trimer with relevance for broadly neutralizing antibody activity
Source: PLoS Biol. 2019 Jan 16;17(1):e3000114. doi: 10.1371/journal.pbio.3000114 (PMC6351000; doi:10.1371/journal.pbio.3000114)
Supplement: S1 Table — Env, envelope glycoprotein. (PDF) [file pbio.3000114.s017.pdf]

S1 Table

Inhibitor	Drug class	Target/Epitope	Reference	Source
**1-79**	Antibody	gp120, V3 crown	Scheid *et al.* 2009 Nature. 458(7238):636-40	M. Nussenzweig,<br>The Rockefeller University, New York, USA
**447-52D**	Antibody	gp120, V3 crown	Gorny *et al.* 1992 J Virol. 66(12):7538-42	H. Katinger and D. Katinger,<br>Polymun, Vienna, Austria
**17b**	Antibody	gp120, CD4i	Thali *et al.* 1993 J Virol. 67(7):3978-88	J. Robinson,<br>Tulane University Medical Center, New Orleans, USA
**48d**	Antibody	gp120, CD4i	Thali *et al.* 1993 J Virol. 67(7):3978-88	J. Robinson,<br>Tulane University Medical Center, New Orleans, USA
**b6**	Antibody	gp120, CD4bs	Barbas III *et al.* 1992 Proc Natl Acad Sci U S A. 89(19):9339-43	D. Burton,<br>The Scripps Research Institute, La Jolla, USA
**b12**	Antibody	gp120, CD4bs	Barbas III *et al.* 1992 Proc Natl Acad Sci U S A. 89(19):9339-43	D. Burton,<br>The Scripps Research Institute, La Jolla, USA
**VRC01**	Antibody	gp120, CD4bs	Wu *et al.* 2010 Science. 329(5993):856-61	J. Mascola*,<br>Vaccine Research Center, National Institutes of Health, Bethesda, USA
**2G12**	Antibody	gp120, high mannose patch	Trkola *et al.* 1996 J Virol. 70(2):1100-8	H. Katinger and D. Katinger,<br>Polymun, Vienna, Austria
**PGT128**	Antibody	gp120, high mannose patch	Walker *et al.* 2011 Nature. 477(7365):466-70	D. Burton,<br>The Scripps Research Institute, La Jolla, USA
**PGT121**	Antibody	gp120, high mannose patch	Walker *et al.* 2011 Nature. 477(7365):466-70	D. Burton,<br>The Scripps Research Institute, La Jolla, USA
**PGT135**	Antibody	gp120, high mannose patch	Walker *et al.* 2011 Nature. 477(7365):466-70	D. Burton,<br>The Scripps Research Institute, La Jolla, USA
**PG9**	Antibody	gp120, trimer apex	Walker *et al.* 2009 Science. 326(5950):285-9	D. Burton,<br>The Scripps Research Institute, La Jolla, USA
**PG16**	Antibody	gp120, trimer apex	Walker *et al.* 2009 Science. 326(5950):285-9	D. Burton,<br>The Scripps Research Institute, La Jolla, USA
**PGT145**	Antibody	gp120, trimer apex	Walker *et al.* 2011 Nature. 477(7365):466-70	D. Burton,<br>The Scripps Research Institute, La Jolla, USA
**PGDM1400**	Antibody	gp120, trimer apex	Sok *et al.* 2014 Proc Natl Acad Sci U S A. 111(49):17624-9	D. Burton,<br>The Scripps Research Institute, La Jolla, USA
**35O22**	Antibody	gp120/gp41 interface	Huang *et al.* 2014 Nature. 515(7525):138-42	J. Mascola*,<br>Vaccine Research Center, National Institutes of Health, Bethesda, USA
**PGT151**	Antibody	gp120/gp41 interface	Falkowska *et al.* 2014 Immunity. 40(5):657-68	D. Burton,<br>The Scripps Research Institute, La Jolla, USA
**VRC34.01**	Antibody	gp41 fusion peptide	Kong *et al.* 2016 Science. 352(6287):828-33	J. Mascola,<br>Vaccine Research Center, National Institutes of Health, Bethesda, USA
**5F3**	Antibody	gp41 FPPR	Buchacher *et al.* 1994 AIDS Res Hum Retroviruses. 10(4):359-69	H. Katinger and D. Katinger,<br>Polymun, Vienna, Austria
**25C2**	Antibody	gp41 FPPR	Buchacher *et al.* 1994 AIDS Res Hum Retroviruses. 10(4):359-69	H. Katinger and D. Katinger,<br>Polymun, Vienna, Austria
**3D6**	Antibody	gp41 cluster I	Buchacher *et al.* 1994 AIDS Res Hum Retroviruses. 10(4):359-69	H. Katinger and D. Katinger,<br>Polymun, Vienna, Austria

S1 Table

**4B3**	Antibody	gp41 cluster I	Buchacher *et al.* 1994 AIDS Res Hum Retroviruses. 10(4):359-69	H. Katinger and D. Katinger,<br>Polymun, Vienna, Austria
**4D4**	Antibody	gp41 cluster I	Buchacher *et al.* 1994 AIDS Res Hum Retroviruses. 10(4):359-69	H. Katinger and D. Katinger,<br>Polymun, Vienna, Austria
**240-D**	Antibody	gp41 cluster I	Xu *et al.* 1991 J Virol. 65(9):4832-8	S. Zolla-Pazner*,<br>Mount Sinai School of Medicine, New York, USA
**50-69**	Antibody	gp41 cluster I	Xu *et al.* 1991 J Virol. 65(9):4832-8	S. Zolla-Pazner*,<br>Mount Sinai School of Medicine, New York, USA
**98-6**	Antibody	gp41 cluster II	Xu *et al.* 1991 J Virol. 65(9):4832-8	S. Zolla-Pazner*,<br>Mount Sinai School of Medicine, New York, USA
**126-7**	Antibody	gp41 cluster II	Xu *et al.* 1991 J Virol. 65(9):4832-8	S. Zolla-Pazner*,<br>Mount Sinai School of Medicine, New York, USA
**167-D IV**	Antibody	gp41 cluster II	Xu *et al.* 1991 J Virol. 65(9):4832-8	S. Zolla-Pazner*,<br>Mount Sinai School of Medicine, New York, USA
**2F5**	Antibody	gp41, MPER	Buchacher *et al.* 1994 AIDS Res Hum Retroviruses. 10(4):359-69	H. Katinger and D. Katinger,<br>Polymun, Vienna, Austria
**4E10**	Antibody	gp41, MPER	Stiegler *et al.* 2001 AIDS Res Hum Retroviruses. 17(18):1757-65	H. Katinger and D. Katinger,<br>Polymun, Vienna, Austria
**10E8**	Antibody	gp41, MPER	Huang *et al.* 2012 Nature. 491(7424):406-12	M. Connors*,<br>National Institute of Allergy and Infectious Diseases, NIH, Bethesda, USA
**C34**	HR2 mimic, fusion inhibitor	gp41, HR1	Chan *et al.* 1998 Proc Natl Acad Sci U S A. 95(26): 15613–15617	Purchased from AnaSpec Inc.
**C34-IgG$_1$**	C34-antibody chimera	gp41, HR1	Si *et al.* 2004 Proc Natl Acad Sci U S A. 101(14):5036-41	A. Herschhorn and J. Sodroski,<br>Department of Immunology Cancer and Virology, Dana-Farber Cancer Institute, Boston, USA
**CD4-IgG$_2$**	CD4-antibody chimera	gp120, CD4bs	Allaway *et al.* 1995 AIDS Res Hum Retroviruses. 11(5):533-9	W. Olson*,<br>Progenics Pharmaceuticals Inc.
**sCD4**	Soluble CD4 protein	gp120, CD4bs	This study	Produced in *E. coli*, see Material and methods

* Through the NIH AIDS Reagent Program, Division of AIDS, NIAID, NIH
