# Supplementary material for: CD4 occupancy triggers sequential pre-fusion conformational states of the HIV-1 envelope trimer with relevance for broadly neutralizing antibody activity
Source: PLoS Biol. 2019 Jan 16;17(1):e3000114. doi: 10.1371/journal.pbio.3000114 (PMC6351000; doi:10.1371/journal.pbio.3000114)
Supplement: S2 Table — (PDF) [file pbio.3000114.s018.pdf]

S2 Table

Plasmid	Description	Cloning vector	Reference	Source
**MN.3**	MN.3 Env<br>expression plasmid	unknown	Burioni *et al.* 2008 PLoS One. 3(10):e3423	J. Mascola,<br>Vaccine Research Center, National Institutes of Health, Bethesda, USA
**BaL.01**	BaL.01 Env<br>expression plasmid	pcDNA3.1D/V5-His-TOPO	Li *et al.* 2006 J Virol. 80(3):1414-26	J. Mascola*,<br>Vaccine Research Center, National Institutes of Health, Bethesda, USA
**BaL.01 SOS****	BaL.01 SOS Env<br>expression plasmid	pcDNA3.1D/V5-His-TOPO	Li *et al.* 2006 J Virol. 80(3):1414-26	J. Mascola*,<br>Vaccine Research Center, National Institutes of Health, Bethesda, USA
**JR-FL**	JR-FL Env<br>expression plasmid	pcDNA3.1/V5-His-TOPO	Rusert *et al.* 2009 AIDS. 23(11):1319-27	N/A
**BG505_T332N****	BG505.W6M.ENV.C2_T332N Env<br>expression plasmid	pcDNA3.1/V5-His-TOPO	Wu *et al.* 2006 J Virol. 80(2):835-44	Julie Overbaugh*,<br>Fred Hutchinson Cancer Research Center, Seattle, USA
**pCMV-rev**	HIV-1 Rev<br>expression plasmid	pCMV	Lewis *et al.* 1990 J Virol. 64(4):1690-7	M. Hammarskjöld and D. Rekosh*,<br>The Scripps Research Institute, La Jolla, USA
**pNL-lucAM**	Partial Δ*env* HIV-1 pseudotyping vector<br>with firefly luciferase reporter gene	pNL4-3	Pugach *et al.* 2007 Virology. 361(1):212-28	A. Marozsan and J. Moore,<br>Weill Medical College, Cornell University, New York, USA
**sCD4-183_avi_his**	Recombinant soluble CD4<br>expression plasmid	pET-32a(+)	This study	N/A
**pBirAcm**	BirA biotin ligase<br>expression plasmid	pACYC-184		Avidity LLC

* Through the NIH AIDS Reagent Program, Division of AIDS, NIAID, NIH

** The SOS mutations (A501C/T605C) and the T332N mutation was introduced with QuikChange II XL Site-Directed Mutagenesis Kit (Agilent Technologies, Santa Clara, USA) according to manufacturer’s instructions
